# Rev–Rev Response Element Activity Selection Bias at the Human Immunodeficiency Virus Transmission Bottleneck

**DOI:** 10.1093/ofid/ofad486

**Published:** 2023-09-29

**Authors:** Patrick E H Jackson, Jordan Holsey, Lauren Turse, Marie-Louise Hammarskjold, David Rekosh

**Affiliations:** Division of Infectious Diseases and International Health, University of Virginia, Charlottesville, Virginia, USA; Myles H. Thaler Center for AIDS and Human Retrovirus Research, University of Virginia, Charlottesville, Virginia, USA; Myles H. Thaler Center for AIDS and Human Retrovirus Research, University of Virginia, Charlottesville, Virginia, USA; Myles H. Thaler Center for AIDS and Human Retrovirus Research, University of Virginia, Charlottesville, Virginia, USA; Myles H. Thaler Center for AIDS and Human Retrovirus Research, University of Virginia, Charlottesville, Virginia, USA; Department of Microbiology, Immunology, and Cancer Biology, University of Virginia, Charlottesville, Virginia, USA; Myles H. Thaler Center for AIDS and Human Retrovirus Research, University of Virginia, Charlottesville, Virginia, USA; Department of Microbiology, Immunology, and Cancer Biology, University of Virginia, Charlottesville, Virginia, USA

**Keywords:** HIV, HIV Rev, viral pathogenesis, viral transmission

## Abstract

**Background:**

Sexual transmission of human immunodeficiency virus (HIV) is inefficient and results in selection of viral variants based on incompletely understood factors. Functional variation in the Rev–Rev response element (RRE) regulatory axis of HIV affect replication kinetics and relative expression of viral proteins. We explored whether differences in this axis among viral isolates affect transmission fitness.

**Methods:**

HIV sequences were identified from nine female-to-male transmission pairs. Using a rapid flow cytometric assay, we analyzed Rev-RRE functional activity of primary isolates.

**Results:**

Rev-RRE activity was significantly lower in recipient viruses compared with corresponding donor viruses. In most transmission events, recipient virus Rev-RRE activity clustered at the extreme low end of the range of donor virus activity.

**Conclusions:**

These data indicate selection pressure on the Rev-RRE axis during female-to-male sexual transmission. Variation in Rev-RRE activity may permit viral adaptation to different fitness landscapes and could play an important role in HIV pathogenesis.

Sexual transmission of human immunodeficiency virus (HIV) is a frequent occurrence worldwide, accounting for >1 million incident infections in 2021 [1]. Paradoxically, the virus is not easily transmitted between hosts. The vast majority of unprotected sexual encounters between serodiscordant partners do not result in a new infection [2]. Even in cases where sexual transmission does occur, of the many viral variants circulating in the donor partner usually only a single virus successfully enters the new host and establishes infection [3, 4]. This sharp reduction in viral diversity between the previously infected donor and the newly infected recipient is termed the *transmission bottleneck*.

A virus must traverse multiple potential barriers to successfully transmit HIV from a female to a male host via penile-vaginal intercourse [5]. The viral quasispecies in the donor's genital compartment may differ from that of the systemic circulation owing to the immune microenvironment, and a subset of genital tissue resident variants may predominate in genital fluids [6]. The recipient's genital mucosa presents a physical obstacle to infection and may also represent an immunologic landscape that differs from that of the donor's genital immune milieu. The virus must then successfully infect a susceptible cell within the host's genital tissue and establish productive infection in a regional lymph node. Only then can systemic dissemination occur. Each of these steps—replication in donor genital tissue, entry into donor transmission fluid, infection of a recipient cell past the mucosa, and establishment of productive infection in recipient lymphoid tissue—may or may not individually represent a significant obstacle to transmission. However, the net effect of the transmission bottleneck exerts selection pressure on transmitted/founder (T/F) variants [7].

The T/F virus is generally not the most predominant variant in the donor genital compartment, as would be anticipated if transmission were a mere stochastic process [8]. The selected phenotype of the T/F variant is incompletely understood. Selection for CCR5 tropism has been consistently demonstrated across studies [3, 9, 10]. Several studies have also observed selection for interferon (IFN) resistance of T/F variants [11–14], though this remains controversial [15, 16]. Phenotypic differences in characteristics such as infectivity [12], virus particle release [11] and envelope content [12] have also been seen in some but not all transmission studies [15, 16]. These conflicting results may be due to differences in assay methods and participant populations. Selection pressures may differ by route of HIV transmission, and factors such as concurrent genital inflammation and high donor viral load may decrease selection stringency [7].

In order to replicate, HIV must overcome the cellular restrictions to the export of intron-containing viral messenger RNAs (mRNAs) from the nucleus to the cytoplasm [17]. This is accomplished by means of a *trans*-acting viral protein, Rev, in conjunction with a *cis*-acting RNA secondary structure, the Rev response element (RRE), found in all the viral mRNAs with retained introns [18, 19]. Rev is constitutively expressed from a completely spliced viral mRNA. After translation in the cytoplasm, the Rev protein is imported into the nucleus, where it binds to the RRE and oligomerizes [20–22]. The RRE-Rev complex then recruits cellular factors, including Crm1 and Ran-GTP, to form a complex capable of exporting the intron-containing viral mRNAs to the cytoplasm for translation or packaging into new viral particles [23].

HIV primary isolates exhibit sequence differences in both *rev* and the RRE, and this in turn results in substantial functional activity variation in the Rev-RRE axis between variants [24, 25]. Small sequence differences in Rev, the RRE, or both can yield significant differences in Rev-RRE axis activity [26, 27]. As the Rev-RRE interaction is necessary for the nuclear export and translation of intron-containing but not fully spliced viral mRNAs, differences in the level of Rev-RRE activity affect not only viral replication kinetics but also the relative expression of many viral proteins [28]. Work on another complex retrovirus with a functionally homologous Rev-RRE system, equine infectious anemia virus, has shown that Rev-RRE activity can vary during the course of chronic infection and that the level of activity correlates with clinical disease state [29, 30]. Some small studies have also proposed that differences in HIV Rev or RRE activity may affect clinical progression [28, 31, 32]. Thus, Rev-RRE activity could be a potential factor that contributes to the phenotype of the T/F virus, but this has not been examined to date.

In the current study, we examined whether functional differences in the Rev-RRE regulatory axis affect variant fitness at the transmission bottleneck. As different selection pressures may influence transmission via different routes [7], we chose to measure differences in Rev-RRE functional activity among primary isolates obtained from individuals in linked female-to-male HIV transmission pairs.

## METHODS

### Sequence Selection and Processing

Single-genome HIV sequences from 18 individuals, consisting of 9 female-to-male transmission pairs, were identified using the Los Alamos HIV Sequence Database (http://www.hiv.lanl.gov/) and GenBank [33]. The sequences were previously published by others [11, 16] (see Table 1, Supplementary Tables 1, and 2 for accession numbers). Only single viral genomes sequenced from plasma that included the RRE and both exons of *rev* were used in this study. RREs and Rev open reading frames that were free of obvious sequencing errors were extracted from the original record (see Supplementary Methods).

For each individual, unique Rev amino acid and unique RRE nucleotide sequence pairs were identified within the set of complete genomic sequences. In addition, unique Rev-RRE cognate pairs (ie, the unique combination of a Rev amino acid sequence and an RRE nucleotide sequence in the same viral genome) were identified. The relative prevalence of unique Revs, RREs, and Rev-RRE pairs within an individual quasispecies was calculated as the number of viral genomes in which this sequence occurred, divided by the total number of viral genomes with intact Rev and RRE sequences in that individual.

All unique Rev-RRE pairs found in ≥12% of circulating variants within an individual quasispecies were included in functional assays. Additional Rev-RRE pairs were selected for functional assays based on Rev or RRE prevalence. No prediction of Rev-RRE functional activity was performed before selecting sequences for inclusion in functional assays. Patient consent was not required for this study as no human subjects research was conducted as part of this work.

### Phylogenetic Analysis

**Figure 1.**
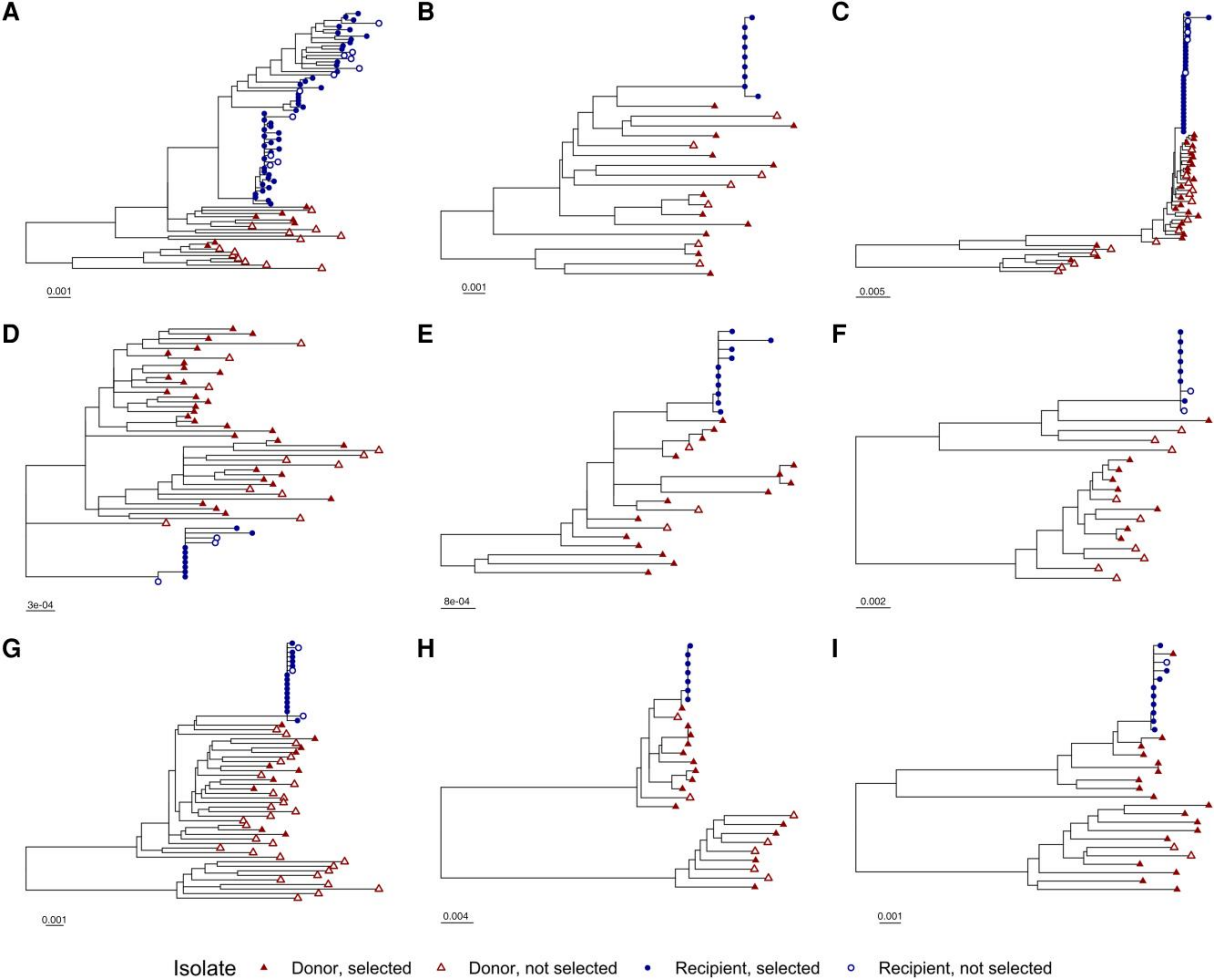
Phylogenetic trees for individual transmission pairs. A phylogenetic tree was generated using the neighbor-joining method for 401 single-genome human immunodeficiency virus (HIV) sequences. The sequences of 4 hundred primary isolates associated with 18 individuals in 9 linked female-to-male HIV transmission pairs were obtained from GenBank. The laboratory strain NL4-3 was included in tree generation as an outgroup but was excluded from the figure display for clarity. Portions of the tree corresponding to the individual transmission pairs, *A–I*, are displayed separately. Tip symbols differentiate sequences from donors and recipients, as well as genomes containing Rev–Rev response element pairs that were selected or not selected for inclusion in functional assays. Horizontal bars represent nucleotide substitutions per site. See also Supplementary Figures 1 and 2.

Phylogenetic trees were generated using the viral genomic sequences listed in Table 1. A neighbor-joining phylogenetic tree was generated using the TreeMaker tool from the Los Alamos HIV database (https://www.hiv.lanl.gov/components/sequence/HIV/treemaker/treemaker.html) and using a Jukes-Cantor distance model with equal site rate. Tree visualizations were created using R software, version 4.2.1, and the package ggtree [34] (Figure 1 and Supplementary Figure 1).

### Functional Assays

Rev-RRE functional activity assays were performed using a flow cytometry-based system that has been described elsewhere [35]. This system includes 2 packageable vector constructs containing Rev or RRE sequences. Additional assay details are available in the Supplementary Methods. The plasmid constructs used in these experiments are listed in Supplementary Table 3.

To perform the functional assays, SupT1 cells were cotransduced with 1 Rev-containing and 1 RRE- containing assay construct. Flow cytometry was performed 72 hours after transduction.

For each experimental run in which a particular Rev-RRE pair was assayed, 3 replicate wells were transduced with the same vector constructs and the mean activity measurement of all interpretable wells for a particular Rev-RRE pair was calculated. Only experimental runs in which ≥2 wells containing a particular Rev-RRE pair were interpretable were used to contribute data for the activity of that pair. A single experimental run including 2 or 3 wells transduced with Rev-RRE pair was considered a single technical replicate for the purposes of statistical analysis.

### Statistical Analysis

Relative Rev-RRE activity was compared between unique cognate pairs using a linear mixed model by restricted maximum likelihood, where an experimental run was considered a random effect and the Rev-RRE pair a fixed effect. Statistical analysis was performed using R software, version 4.1.2, and the lme4 [36] and lmerTest packages [37]. Activity estimates for each unique cognate pair were expressed as a multiple of the activity of the NL4-3 Rev-NL4-3 RRE cognate pair.

To compare Rev-RRE activity between all donor and recipient quasispecies across all transmission pairs, the lme4 package was used to model variant activity with donor versus recipient status as a fixed effect and transmission pair as a random effect. Rev-RRE pair activity values were weighted according to the frequency of occurrence within an individual quasispecies. The estimated marginal means were then calculated and compared for donors versus recipients, using R software, version 4.1.2, and the emmeans package [38]. To compare Rev-RRE activity between donor and recipient quasispecies within a particular transmission pair, the activity level of unique Rev-RRE pairs was weighted by prevalence, and an independent-samples Mann-Whitney *U* test was performed.

To compare the activity of Rev-RRE cognate pairs and corresponding artificial pairs, activity measurements for each Rev-RRE pair were normalized to the activity of the NL4-3 Rev-RRE cognate pair that was included in the same experimental run. Analysis of the difference between cognate pair activity and the activity of the corresponding NL4-3 Rev/primary RRE and primary RRE/NL4-3 Rev pairs was conducted using a 1-way analysis of variant test, with adjustment for multiple comparisons using the Dunnett T3 method.

## RESULTS

### Identification of Rev-RRE Sequences

We selected HIV-1 single-genome sequences from 18 individuals in 9 linked female-to-male HIV transmission pairs (Table 1). Eight individuals (4 transmission pairs) were participants in the Center for HIV/AIDS Vaccine Immunology (CHAVI-001) acute infection cohort [11, 39], and 10 individuals (5 transmission pairs) were participants in the Zambia-Emory HIV Research Project [16]. All sequences were previously published in GenBank (https://www.ncbi.nlm.nih.gov/genbank/). Each HIV transmission pair was given a code, from A through I. Recipient samples were obtained during acute infection (Fiebig stage 4 or earlier). The time elapsed between the acquisition of the donor and recipient samples was a maximum of 265 days (median, 19 days). All viruses were subtype C.

**Table 1.** Sources of Human Immunodeficiency Virus Genome Sequences

Pair	Partner	Time Between Donor and Recipient Samples, d	Fiebig Stage	No. of Single HIV Genomes	No. of Unique Rev Amino Acid Sequences	No. of Unique RRE Nucleotide Sequences	No. of Unique Rev-RRE Pairs	Sequence Accession Nos.	Participant Code in Reference	Reference
A	Donor	19	…	20	9	12	17	KY112094–KY112126	CH0212	Iyer et al [11]
	Recipient		3	60	8	7	14	JX972986–JX972998, JX973019–JX973074	CH0162	
B	Donor	6	…	18	9	16	17	KR820367–KR820384	Z3678F	Deymier et al [16]
	Recipient		2	9	1	2	2	KR820385–KR820393	Z3678M	
C	Donor	47	…	38	15	16	23	KY112322–KY112359	CH0492	Iyer et al [11]
	Recipient		1–2	33	2	5	6	KY112218–KY112250	CH0427	
D	Donor	265	…	41	6	9	14	KY112015–KY112055	CH1064	Iyer et al [11]
	Recipient		4	12	2	3	4	KX216883–KX216893, KX216895	CH0848	
E	Donor	0	…	18	8	4	12	KR820422–KR820439	Z4473F	Deymier et al [16]
	Recipient		2	10	1	2	2	KR820440–KR820449	Z4473M	
F	Donor	23	…	17	8	8	13	KR820341–KR820357	Z3618F	Deymier et al [16]
	Recipient		2	9	2	2	3	KR820358–KR820366	Z3618M	
G	Donor	65	…	39	13	27	33	KY112390–KY112428	CH0596	Iyer et al [11]
	Recipient		3	18	3	2	4	KY111965–KY111982	CH0455	
H	Donor	7	…	21	8	14	16	KR820394–KR820414	Z4248F	Deymier et al [16]
	Recipient		2	7	1	1	1	KR820415–KR820421	Z4248M	
I	Donor	3	…	20	7	11	15	KR820294–KR820313	Z331F	Deymier et al [16]
	Recipient		2	10	2	1	2	KR820314–KR820323	Z331M	

Abbreviations: HIV, human immunodeficiency virus; RRE, Rev response element.

A total of 400 single-genome sequences from these individuals that included the full coding region of *rev* as well as the RRE were analyzed. Phylogenetic trees were generated using the neighbor-joining method to confirm the pattern of HIV transmission (Figure 1). Within each transmission pair, sequences from the recipient clustered together and mostly separately from donor sequences. As expected, sequences from different transmission pairs were not interspersed (Supplementary Figure 1).

The 400 analyzed viral genomic sequences included 105 unique Rev amino acid sequences and 142 unique RRE nucleotide sequences. Some RRE and Rev sequences were observed to occur in multiple primary isolates and in multiple combinations. In all, 198 unique Rev-RRE cognate pairs (ie, a Rev amino acid sequence and an RRE nucleotide sequence in a single viral genome) were identified. Donor quasispecies exhibited more sequence diversity than recipient quasispecies. Donor quasispecies had a median of 16 unique Rev-RRE cognate pairs, while recipient quasispecies had a median of 3 unique cognate pairs.

From the set of 198 unique Rev-RRE cognate pairs, a subset was selected for functional analysis based on prevalence within the participant quasispecies. All Rev-RRE cognate pairs present in ≥12% of viral variants circulating in a single host were included in functional assays. Additional Rev-RRE pairs were included to ensure the highest prevalence Rev and RRE sequences in an individual were represented in functional assays. All in all, a total of 81 unique Rev-RRE cognate pairs were used in the functional activity assays. Of the original 400 analyzed primary isolates, 281 primary isolates contained a Rev-RRE cognate pair that was represented in the functional assays. One Rev-RRE cognate pair occurred in both the donor and recipient in transmission pair E (Supplementary Figure 2). All other Rev-RRE cognate pairs occurred in only a single individual.

**Figure 2.**
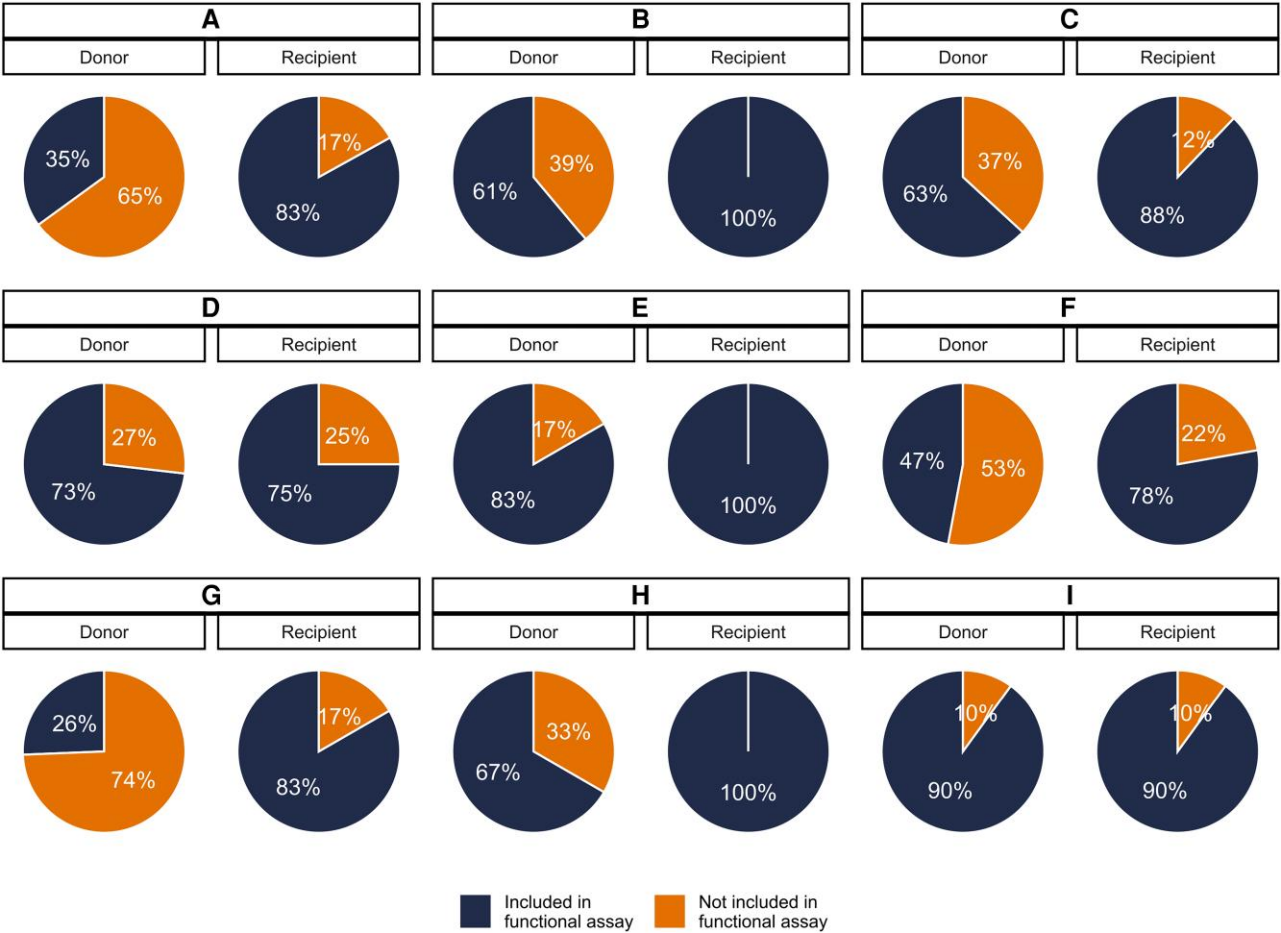
Proportion of viral variants represented in functional activity assays. A subset of the unique Rev–Rev response element (RRE) cognate pairs from primary isolates were included in the functional activity assays. For each individual, the proportion of the sequenced viral variants from that individual's plasma containing a Rev–RRE sequence included in the functional assays is shown. Individuals are indicated by the transmission pair, *A* through *I*, and the position within each transmission pair, either the donor or recipient partner.

Between 1 and 13 unique Rev-RRE cognate pairs were selected for each individual, representing coverage varying between 26% and 100% of the sequenced quasispecies in each individual (Figure 2 and Supplementary Figures 3 and 4). For individuals in whom a few Rev-RRE cognate pairs occurred many times in the quasispecies, greater coverage was accomplished than when many cognate pairs were present at a low frequency (Supplementary Table 1). For the donor individuals in transmission pairs A, F, and G, a minority of the circulating variants were represented in the functional assays, but all Rev-RRE cognate pairs appearing in the quasispecies more than once were included.

### Rev-RRE Functional Activity of Donor and Recipient Viruses

**Figure 3.**
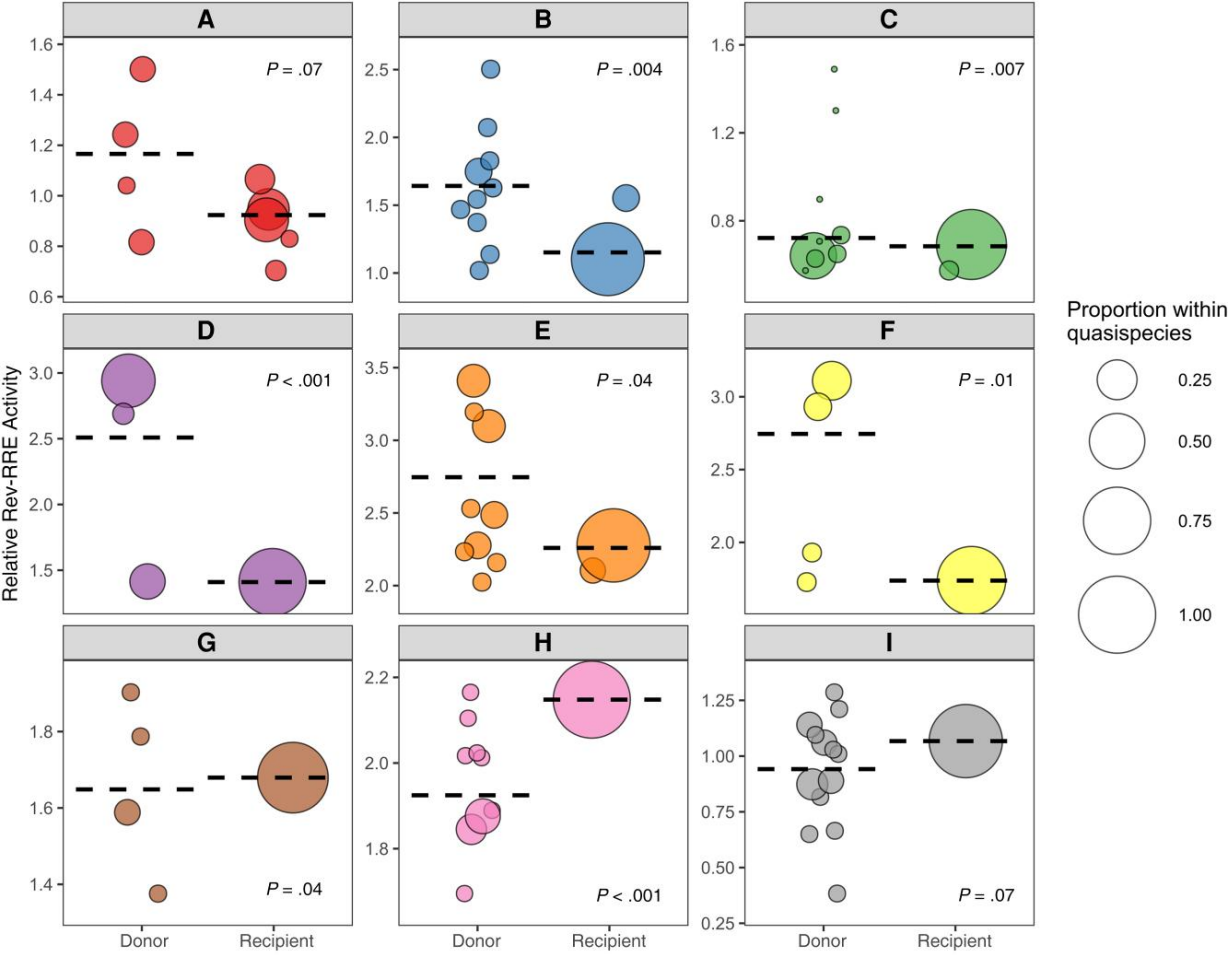
Rev–Rev response element (RRE) functional activity of viral variants from donors and recipients. The relative functional activity of selected Rev-RRE pairs from primary isolates in both donors and recipients is shown. Each human immunodeficiency virus transmission pair is displayed, from *A* through *I*, with Rev-RRE cognate pairs from donor viral sequences on the left of each box and Rev-RRE cognate pairs from recipient viral sequences on the right. Each bubble is a unique Rev-RRE pair in the indicated individual's quasispecies. The position of the bubble on the y-axis represents the relative level of Rev-RRE functional activity for that pair. The area of each bubble is scaled to the relative prevalence of the Rev-RRE pair sequence within the individual's sequenced quasispecies. The weighted average of variant Rev-RRE activity for each individual quasispecies is indicated by the dashed horizontal line. Rev-RRE activity distributions of the donor and the recipient variants were compared for each transmission pair by means of independent-samples Mann-Whitney *U* test, and the *P* value for this comparison is shown in each plot. Activity units are multiples of the Rev-RRE activity of the laboratory strain NL4-3.

The relative functional activity of the selected Rev-RRE cognate pairs was determined using a lentiviral vector-based assay as described elsewhere (see Methods) [27, 35]. As shown in Figure 3, there was an almost 9-fold difference in functional activity between the most-active and least-active Rev-RRE cognate pairs. For each transmission pair where multiple recipient Rev-RRE sequences were assayed, the range of Rev-RRE activity of the donor-derived variants was greater than the range of activity for variants from the corresponding recipient (see also Supplementary Table 1).

Overall, the Rev-RRE activity of recipient-derived variants was significantly lower than the activity of the corresponding donor variants (*P* = .02). In 6 of the 9 transmission pairs, the Rev-RRE activity of the recipient variants clustered at the extreme low end of the range of activity of the corresponding donor variants. This pattern was consistent for transmission pairs with overall high Rev-RRE activity (pairs D, E, F, G, and H) and transmission pairs with overall low Rev-RRE activity (pairs A, B, C, and I). There was no transmission pair where a recipient variant had the highest overall Rev-RRE activity. For transmission pair H, the highest activity donor variant was slightly more active than the recipient variant (2.17 vs 2.15).

In only 3 of the 9 transmission pairs—G, H, and I—was the weighted average of recipient variant Rev-RRE activity greater than the average donor variant activity. It is notable that one of these transmission pairs, I, included the variant with the lowest overall Rev-RRE activity; and the maximum activity of the pair I variants was lower than for any other transmission pair (Supplementary Figure 5). While the weighted average of activity was lowest for transmission pair C, pair C included individual donor variants with higher activity than any variant from pair I.

### Rev and RRE Contributions to Cognate Pair Activity

**Figure 4.**
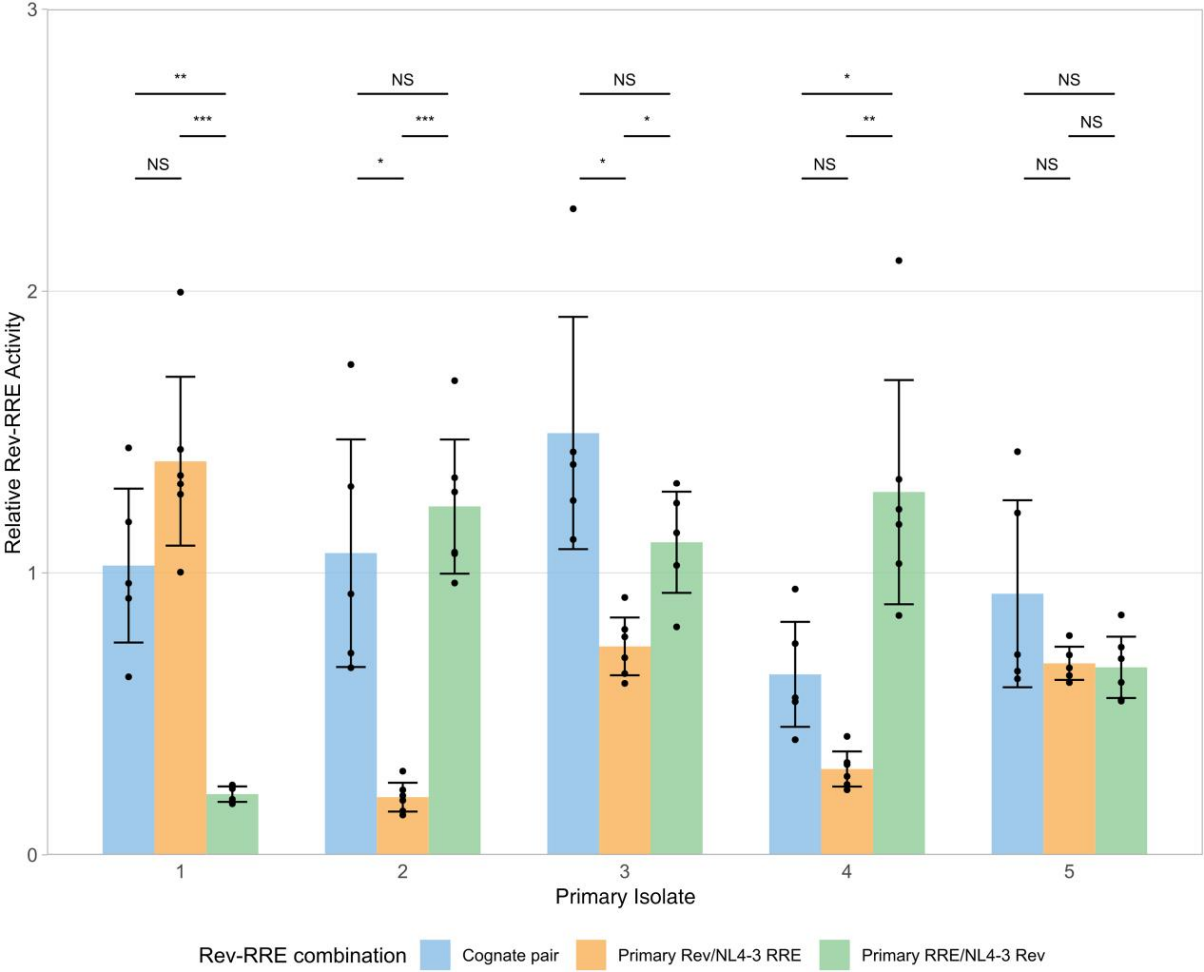
Contributions of Rev and the Rev–response element (RRE) to cognate pair functional activity. The relative functional activity of Rev-RRE cognate pairs from primary isolates, as well as the activity of the component Rev with NL4-3 RRE and the component RRE with NL4-3 Rev is shown. Relative activity is shown as multiples of the activity of the NL4-3 cognate pair without units, such that 1 corresponds to the NL4-3 Rev/NL4-3 RRE pair. Observations from technical replicates (n = 5 or 6) are shown as dots. Bars represent the mean values of individual observations, and error bars represent standard deviation. Statistical comparisons were performed using 1-way analysis of variant with adjustment for multiple comparisons using the Dunnett T3 method. Primary isolate 1 was obtained from donor I (accession no. KR820312), isolate 2 from recipient B (KR820385), isolate 3 from donor G (KY112428), isolate 4 from donor C (KY112346), and isolate 5 from recipient A (JX973051). Cognate pair activity values replicate values shown in Figure 3. Abbreviation: NS, not significant. *P < .05; **P < .01; ***P < .001.

To evaluate the contribution of Rev and RRE differences to cognate pair activity, artificial combinations of Revs and RREs were next tested in the functional assay. Artificial Rev-RRE pairs consisted of a Rev sequence from the HIV laboratory strain NL4–3 paired with an RRE from a primary isolate or a Rev from a primary isolate paired with the NL4-3 RRE. For 5 primary isolate Rev-RRE cognate pairs, functional assays were performed comparing the activity of the primary isolate cognate pairs with these artificial pairs (Figure 4).

Cognate pair functional activity could not be predicted by component Rev or RRE activity. For example, in primary isolate 1, the combination of the NL4-3 RRE with the isolate Rev yielded numerically higher activity than the original cognate pair, while pairing NL4-3 Rev with the isolate RRE yielded significantly lower activity. The converse was observed for primary isolate 2. This is consistent with previous results indicating that changes in either Rev or the RRE are sufficient to significantly alter Rev-RRE axis activity.

## DISCUSSION

In the current study, we observed a previously unreported selection based on the Rev-RRE axis during female-to-male sexual transmission of HIV. Globally, recipient viral variants displayed lower Rev-RRE activity than variants from corresponding donors, and in 6 of 9 transmission events recipient variant activity clustered at the extreme low end of the range of activity of the corresponding donor variants. Recipient variant Rev-RRE activity was generally not the same as the activity of the predominant variant in the donor plasma compartment. These results are most consistent with the female-to-male sexual transmission bottleneck, conferring a selection advantage for viruses with a lower level of Rev-RRE activity.

There were 3 discordant observations, however, where the weighted average of recipient variant Rev-RRE activity was higher than that of the corresponding donor. For transmission pair I, the range of Rev-RRE activity for variants from both the donor and recipient was low compared with the set of variants from all transmission pairs. If Rev-RRE selection at the transmission bottleneck is subject to a threshold effect in particular individuals, then the activity of the donor viruses may have been sufficiently low that there was no additional selection on the variants during transmission. While this potential explanation would not account for transmission pairs G and H, in which variants displayed intermediate levels of Rev-RRE activity, selection pressures at the transmission bottleneck may be mitigated by conditions that predispose to forward transmission, including concurrent genital inflammation [7] and immune compromise. In the absence of additional clinical data for these study participants, it is unclear whether these factors could account for the cases where recipient Rev-RRE activity was higher.

The pattern of donor and recipient virus Rev-RRE activity is unlikely to be secondary to selection for other phenotypic parameters, such as coreceptor utilization or IFN resistance. As demonstrated previously and again in this study, Rev-RRE activity is highly sensitive to changes in the RRE, *rev*, or both. This permits a high degree of plasticity in the regulatory axis and an ability to accommodate extrinsic pressures while maintaining Rev-RRE activity level. Previous work by others has demonstrated that functional regions of *rev* are segregated from functional regions of the overlying *tat* and *env* genes, permitting mutations that affect the activity of one protein at a time [40, 41]. RRE activity is similarly robust to nonsynonymous changes in the envelope (Env), as the functional consequence of these mutations can be compensated by additional synonymous changes in other portions of the RRE [42, 43]. Therefore, a constraint on Env sequence imposed by a selection for type I IFN resistance at the transmission bottleneck, for example, would not be expected to impose a constraint on T/F Rev-RRE activity level. Indeed, Iyer et al [11] experimentally demonstrated widely disparate levels of IFN-α2 and IFN-β sensitivity among these primary isolates, some of which share identical Rev-RRE cognate pairs.

An analysis of larger numbers of female-to-male transmission pairs are necessary to validate the finding of apparent selection pressure on the Rev-RRE axis described here. Future studies should ideally also include viral variants of different subtypes since these may be subject to different selection pressures [12]. Other routes of HIV transmission, such as male-to-female sexual transmission, transmission via insertive or receptive anal intercourse, vertical transmission, and parenteral transmission may also create alternate selection pressures. In this study, we did not assess other viral factors that may contribute to transmission fitness, such as replication capacity, but this may be affected directly by Rev-RRE activity. A more complete phenotypic characterization of T/F variants may yield insights into the optimal constellation of viral characteristics that facilitate sexual transmission.

In this study, we conceptualized the transmission bottleneck as the sum total effect of multiple potential immunologic and anatomic barriers to the sexual transmission of HIV. We did not assess the contribution of individual potential barriers to selection pressure on the T/F virus. The selection we observe could occur entirely within the donor or the recipient, or selection could be an emergent phenomenon of processes within both hosts. As in other clinical studies of HIV transmission, the single T/F virus was not directly sampled, and viral sequences from the recipient close to the time of transmission were used as a proxy for determining the characteristics of the T/F variant. The limited genetic and functional diversity of recipient viruses suggests that this approach was successful in the present study. However, we cannot exclude rapid selection on the Rev-RRE system occurring during early dissemination in the new host accounting for these findings, rather than selection occurring during the initial sexual transmission.

While we did not examine the mechanism by which Rev-RRE activity selection occurs during transmission, 2 factors may yield a selective advantage for variants with different levels of Rev-RRE functional activity. First, the Rev-RRE axis affects viral replication capacity. We previously demonstrated with replication-competent HIV constructs that Rev activity is positively correlated with replication kinetics [27]. Second, Rev-RRE activity alters the relative expression of viral proteins encoded by completely spliced mRNA species (ie, Tat, Rev, and Nef) to proteins encoded by mRNAs with retained introns (eg, Gag, Env, Vpr) [28].

These factors may alter transmission fitness in various ways. Viral replication and Vpr production is associated cytopathic effects [44], and this may be mitigated to some extent by lower Rev-RRE activity in the infecting virus. Cell-associated virus may play a key role in transmission [45], so the extent of cytotoxicity induced by a variant may influence its ability to establish a new infection. In addition, Nef modulates the immune response to HIV infection by down-regulating CD4 and major histocompatibility complex class 1 expression on the surface of infected cells [46]. By maintaining a protective level of Nef expression (independent of Rev-RRE function) and lower expression of the Rev-RRE dependent structural proteins that generate antigenic peptides, viruses with low Rev-RRE functional activity appear to be relatively protected from cytotoxic T-cell–mediated killing [28]. An immune evasive strategy could provide a selective advantage for variants in donor genital tissues or during early disseminated infection in the recipient. The ability of Nef to mitigate NK-cell mediated responses [47] and to modulate dendritic cell function [48] may also help explain the selective advantage for variants with lower Rev-RRE activity.

The current study sheds light on 2 areas of investigation. First, we suggest Rev-RRE activity as a new factor in phenotypic selection at the HIV transmission bottleneck. If additional studies confirm this finding, accounting for Rev-RRE activity variation may help to reconcile the currently conflicting data on viral transmission fitness and could potentially point to new strategies for transmission prevention. Second, this study adds to the literature suggesting a role for variation in the Rev-RRE system in HIV pathogenesis [49] and strengthens the concept that the Rev-RRE axis can function as a molecular rheostat to allow the virus to adapt to different pressures. This paradigm may be important not only in HIV transmission but also in other processes where viral adaptation to differing immune environments could affect pathogenesis, including viral compartmentalization and latency.

## Supplementary Material

ofad486_Supplementary_Data

## Supplementary Data

Supplementary materials are available at *Open Forum Infectious Diseases* online (https://academic.oup.com/ofid). Supplementary materials consist of data provided by the author that are published to benefit the reader. The posted materials are not copyedited. The contents of all supplementary data are the sole responsibility of the authors. Questions or messages regarding errors should be addressed to the author.

## Notes

***Author contributions.*** P. E. H. J. contributed to conceptualization of the study, performed functional assays, and wrote the first draft of the manuscript. J. H. and L. T. performed functional assays. M. L. H and D. R. were involved in the conceptualization of the studies and the experimental design and critically revised the manuscript. All authors reviewed the final manuscript.

***Data availability.*** Sequence data are available from GenBank at the accession numbers listed in the text and in Table 1. Additional study data are available from the corresponding author on reasonable request.

***Financial support***. This work was supported by the National Cancer Institute (grant P30-CA044579 supporting flow cytometry experiments), the National Institutes of Health (grants CA206275, AI136671, and AI134208 supporting research on HIV Rev and grant K08 AI136671 providing partial salary support for P. E. H. J.), and the University of Virginia (Myles H. Thaler Research Support Gift, the Myles H. Thaler Professorship [D. R.], and the Charles H. Ross Jr Professorship [M. L. H.]).
