# Supplementary material for: Rev–Rev Response Element Activity Selection Bias at the Human Immunodeficiency Virus Transmission Bottleneck
Source: Open Forum Infect Dis. 2023 Sep 29;10(10):ofad486. doi: 10.1093/ofid/ofad486 (PMC10580148; doi:10.1093/ofid/ofad486)
Supplement: ofad486_Supplementary_Data [file ofad486_supplementary_data.zip › OFID revision supplemental methods and figures.pdf]

Rev-Rev Response Element Activity Selection Bias at the HIV Transmission Bottleneck

Patrick E. H. Jackson[1,2], Jordan Holsey[2], Lauren Turse[2], Marie-Louise Hammarskjold[2,3], David Rekosh[2,3]

1. Division of Infectious Diseases and International Health, University of Virginia, Charlottesville, Virginia, USA
2. Myles H. Thaler Center for AIDS and Human Retrovirus Research, University of Virginia, Charlottesville, Virginia, USA
3. Department of Microbiology, Immunology, and Cancer Biology, University of Virginia, Charlottesville, Virginia, USA

Corresponding author:

Patrick E. H. Jackson
345 Crispell Drive
MR6 2524
Charlottesville, Virginia, USA 22908
1-434-982-3559
pej9j@uvahealth.org

Supplemental Methods.

Sequence selection and processing.

Single genome HIV sequences from eighteen individuals consisting of nine female-to-male transmission pairs were identified using the Los Alamos HIV Sequence Database (http://www.hiv.lanl.gov/) and GenBank [1]. The sequences were previously published by others) [2, 3] (see Tables 1 and S2 for accession numbers). Only single viral genomes sequenced from plasma that included the RRE and both exons of *rev* were utilized in this study.

RRE and *rev* nucleotide sequences were extracted from the original sequence record using the Gene Cutter tool from the Los Alamos HIV database (https://www.hiv.lanl.gov/content/sequence/GENE_CUTTER/cutter.html). Additional sequence analysis and manipulation was performed using Geneious Prime (Dotmatics). Sequences were assessed for clear errors (*i.e.* a premature stop codon at position <100 in *rev,* stop codons in all three forward reading frames within the RRE) and were excluded from further analysis if either of these conditions were met. Rev open reading frames were extracted from the Gene Cutter output.

For each individual, unique Rev amino acid and unique RRE nucleotide sequence pairs were identified within the set of complete genomic sequences. Additionally, unique Rev-RRE cognate pairs (that is, the unique combination of a Rev amino acid sequence and an RRE nucleotide sequence in the same viral genome) were identified. The relative prevalence of unique Revs, RREs, and Rev-RRE pairs within an individual's quasispecies was calculated as the number of

viral genomes in which this sequence occurred, divided by the total number of viral genomes with intact Rev and RRE sequences in that individual.

All unique Rev-RRE pairs found in at least 12% of circulating variants within an individual quasispecies were included in functional assays. Additional Rev-RRE pairs were selected for functional assays based on Rev or RRE prevalence. No prediction of Rev-RRE functional activity was performed prior to selecting sequences for inclusion in functional assays.

Phylogenetic analysis.

Phylogenetic trees were generated using the viral genomic sequences listed in Table 1. Sequences were aligned in Geneious Prime using the Clustal Omega 1.2.2 algorithm [4]. The sequence of the laboratory HIV strain NL4-3 (GenBank accession U26942.1) was included as an outgroup [5]. A neighbor-joining phylogenetic tree was generated using the TreeMaker tool from the Los Alamos HIV database (https://www.hiv.lanl.gov/components/sequence/HIV/treemaker/treemaker.html) utilizing a Jukes-Cantor distance model with equal site rate. Tree visualizations were created using R version 4.2.1 and the package ggtree [6] (Figures 1, S1). The NL4-3 tip was removed from tree visualizations for clarity.

Functional assays.

Rev-RRE functional activity assays were performed using a flow cytometry-based system that has been previously described [7]. This system includes two packageable vector constructs. The first construct is an NL4-3-derived HIV construct with modifications to render it replication incompetent and to silence native *rev* expression. The construct expresses an eGFP fluorescent marker from the *gag* open reading frame in a Rev-RRE dependent fashion and an mCherry fluorescent marker from the *nef* open reading frame in a Rev-RRE independent fashion. The RRE sequences are flanked by restriction sites for exchange in the native position within *env*. The second assay construct is derived from a murine stem cell virus (MSCV) vector. This construct is modified to express an exchangeable Rev along with an eBFP2 fluorescent marker from a bicistronic construct. The plasmid constructs utilized in these experiments are listed in Table S3.

Selected Rev and RRE sequences were commercially synthesized and cloned into MSCV and HIV vector constructs, respectively. The constructs were then packaged and pseudotyped with VSV-G in 293T/17 cells. To perform the functional assays, SupT1 cells were co-transduced with one Rev- and one RRE- containing assay construct. Transductions were performed in 96 well plates with 2.5 x 10^5 SupT1 cells in each well. Cells were transduced at a target multiplicity of infection (MOI) of 0.18. Transductions were performed by combining cells, vector stocks, and 8 mcg/mL DEAE-dextran, and then centrifuging the cultures at 380 RCF for 1 hour at room temperature. Flow cytometry was performed 72 hours after transduction using an Attune NxT flow cytometer with autosampler (Thermo Fischer Scientific). Post-acquisition color compensation and data analysis was performed using FlowJo v10.6.1 (FlowJo, LLC).

To analyze flow cytometry data, gates were constructed to define a single cell population. Next, a gate was constructed to include only cells successfully co-transduced with both the Rev- and RRE-containing assay constructs and expressing both mCherry and eBFP2. In this final population, the mean fluorescence intensity (MFI) of eGFP and eBFP2 was determined. Relative Rev-RRE activity was calculated as the ratio of eGFP to eBFP2 MFI for each well (Figure S6).

For each experimental run in which a particular Rev-RRE pair was assayed, three replicate wells were transduced with the same vector constructs. Individual wells were excluded from analysis if more than 32% of cells were positive for either vector construct or if fewer than 500 cells were successfully co-transduced with both constructs. For every experimental run, the mean activity measurement of all interpretable wells for a particular Rev-RRE pair was calculated. Only experimental runs in which at least two wells containing a particular Rev-RRE pair were interpretable were used to contribute data for the activity of that pair. A single experimental run including two or three wells transduced with Rev-RRE pair was considered a single technical replicate for the purposes of statistical analysis.

Statistics.

The relative functional activity of Rev-RRE pairs was consistent across experimental runs, but the absolute value of the eGFP:eBFP2 ratio used as the measurement of activity level varied between experimental runs presumably due to differences in cell line passage. Relative Rev-RRE activity was compared between unique cognate pairs as in Figure 3 using a linear mixed model by restricted maximum likelihood where an experimental run was considered as a random effect and Rev-RRE pair as a fixed effect. Statistical analysis was performed using R version 4.1.2 and the lme4 [8] and lmerTest packages [9]. Relative Rev-RRE cognate pair activity was calculated from all available experimental runs, and a minimum of three experimental runs (i.e. three technical replicates) was included for each pair in the model. The resulting estimate of Rev-RRE activity for each cognate pair was then expressed as a multiple of the activity estimate for the NL4-3 cognate pair.

To compare Rev-RRE activity between all donor and recipient quasispecies across all transmission pairs, the lme4 package was used to model variant activity with donor vs recipient status as a fixed effect and transmission pair as a random effect. Rev-RRE pair activity values were weighted according to the frequency of occurrence within an individual quasispecies. The estimated marginal means were then calculated and compared for donors versus recipients using R version 4.1.2 and emmeans package [10].

To compare the distribution of Rev-RRE activity between donor and recipient quasispecies within each transmission pair, the activity level of the unique Rev-RRE pairs was first weighted by the frequency with which the Rev-RRE pair was observed in the individual quasispecies. Then the difference between recipient and donor Rev-RRE activity was assessed using the independent samples Mann-Whitney U test in SPSS (IBM). No adjustment for multiple comparisons was made.

To compare the activity of Rev-RRE cognate pairs and corresponding artificial pairs including either the NL4-3 Rev or the NL4-3 RRE as shown in Figure 4, activity measurements for each Rev-RRE pair were normalized to the activity of the NL4-3 Rev-RRE cognate pair that was included in the same experimental run. Analysis of the difference between cognate pair activity and the activity of the corresponding NL4-3 Rev/primary RRE and primary RRE/NL4-3 Rev pairs was conducted using a one-way ANOVA test with adjustment for multiple comparisons using Dunnett's T3 method. This statistical analysis was performed using SPSS (IBM).

1. Benson DA, Cavanaugh M, Clark K, et al. GenBank. Nucleic acids research **2012**; 41:D36-D42.
2. Iyer SS, Bibollet-Ruche F, Sherrill-Mix S, et al. Resistance to type 1 interferons is a major determinant of HIV-1 transmission fitness. Proceedings of the National Academy of Sciences **2017**; 114:E590-E9.

3. Deymier MJ, Ende Z, Fenton-May AE, et al. Heterosexual transmission of subtype C HIV-1 selects consensus-like variants without increased replicative capacity or interferon-α resistance. PLoS Pathog **2015**; 11:e1005154.
4. Sievers F, Higgins DG. Clustal Omega for making accurate alignments of many protein sequences. Protein Science **2018**; 27:135-45.
5. Salminen MO, Koch C, Sanders-Buell E, et al. Recovery of virtually full-length HIV-1 provirus of diverse subtypes from primary virus cultures using the polymerase chain reaction. Virology **1995**; 213:80-6.
6. Yu G, Smith DK, Zhu H, Guan Y, Lam TTY. ggtree: an R package for visualization and annotation of phylogenetic trees with their covariates and other associated data. Methods in Ecology and Evolution **2017**; 8:28-36.
7. Jackson PE, Huang J, Sharma M, Rasmussen SK, Hammarskjold M-L, Rekosh D. A novel retroviral vector system to analyze expression from mRNA with retained introns using fluorescent proteins and flow cytometry. Scientific reports **2019**; 9:1-14.
8. Bates D, Mächler M, Bolker B, Walker S. Fitting Linear Mixed-Effects Models Using lme4. Journal of Statistical Software **2015**; 67:1 - 48.
9. Kuznetsova A, Brockhoff PB, Christensen RH. lmerTest package: tests in linear mixed effects models. Journal of statistical software **2017**; 82:1-26.
10. Lenth R, Singmann H, Love J, Buerkner P, Herve M. Emmeans: Estimated marginal means, aka least-squares means. R Package Version 1 (2018), **2021**.

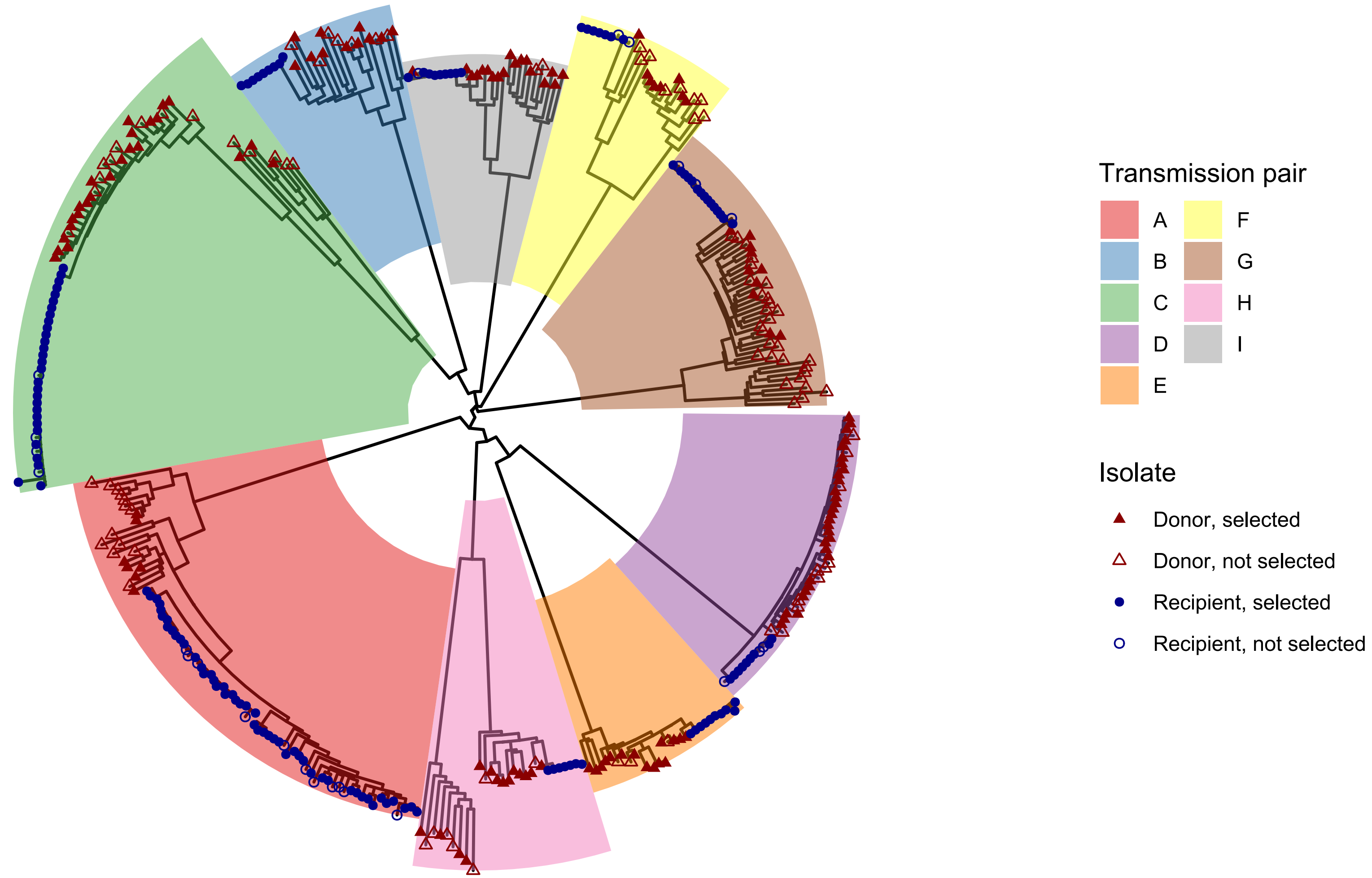

Figure S1. Phylogenetic tree of single genome HIV sequences from eighteen individuals. A phylogenetic tree was generated using the neighbor joining method for 401 single genome HIV sequences. The sequences of four hundred primary isolates associated with eighteen individuals in nine linked female-to-male HIV transmission pairs were obtained from GenBank. The laboratory strain NL4-3 was included in tree generation as an outgroup but was excluded from the figure display for clarity. Branches corresponding to each transmission pair, A through I, are differentiated by colored fields. Tip symbols differentiate sequences from donors and recipients, as well as genomes containing Rev-RRE pairs that were selected or not selected for inclusion in functional assays.

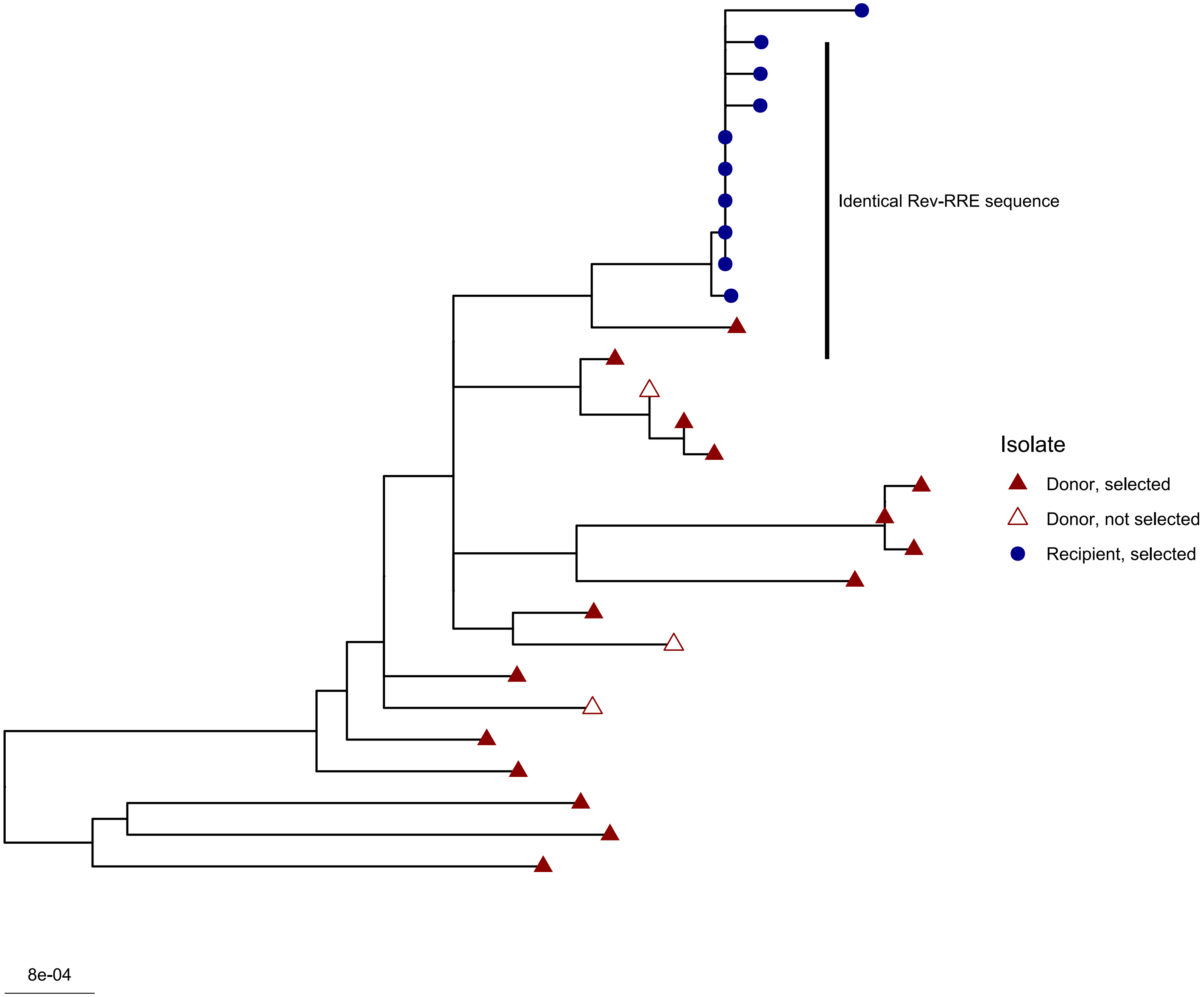

Figure S2. Common Rev-RRE sequence occurring in pair E donor and recipient variants. A phylogenetic tree of the transmission pair E primary isolates was constructed as for Figure 1. Uniquely in this transmission pair, a common Rev-RRE cognate pair is shared between both donor and recipient primary isolates. The nine recipient and two donor primary isolates sharing a common Rev-RRE cognate pair are indicated by the vertical black line. Some branches of this tree are flipped relative to Figure 1 for clarity of presentation.

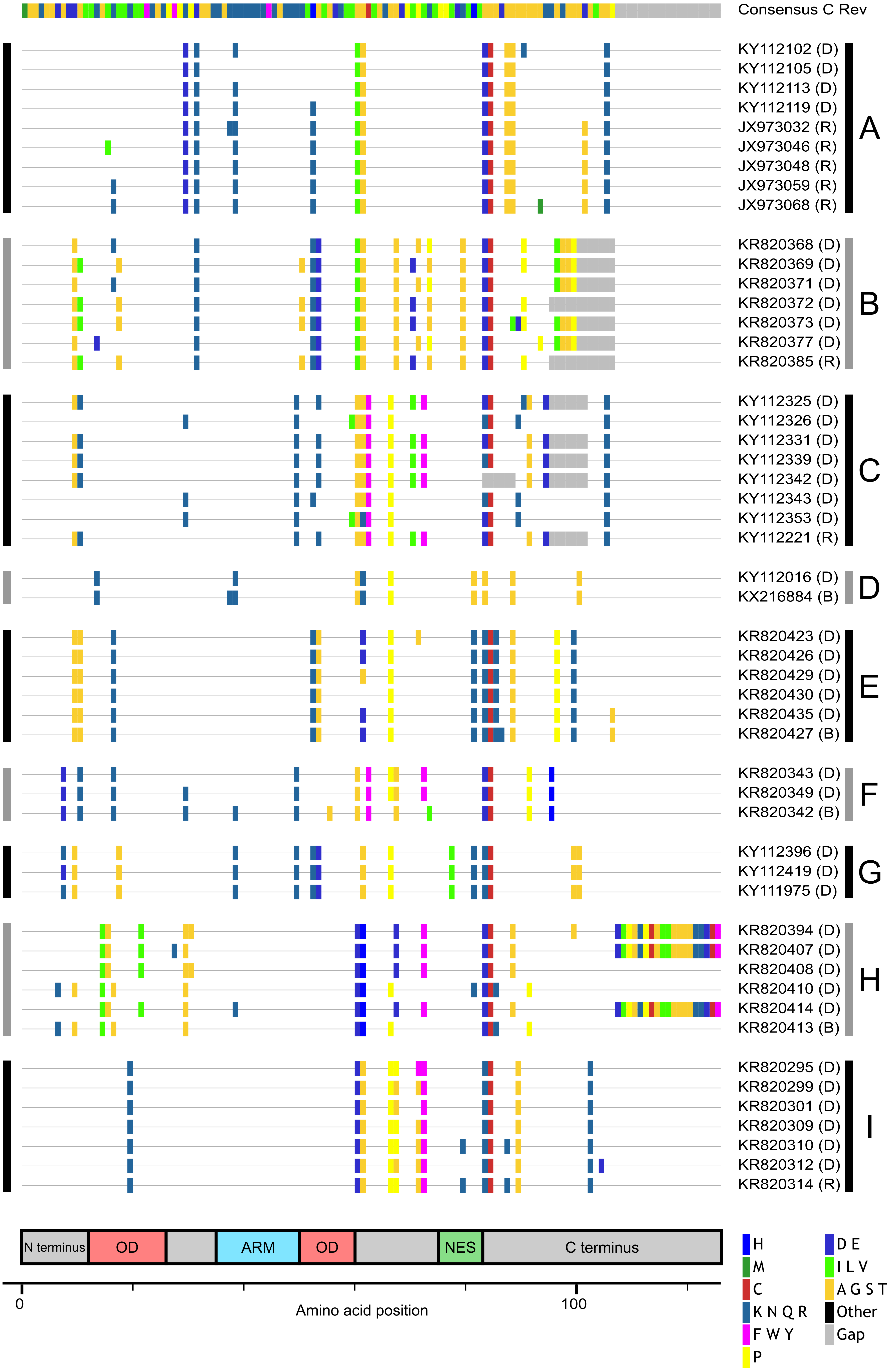

Figure S3. Alignment of unique Rev sequences included in functional assays. Fifty-one unique Rev amino acid sequences from primary isolates that were included in functional assays were aligned along with the Rev from a subtype C consensus sequence (Los Alamos HIV database 2002 consensus, https://www.hiv.lanl.gov/content/sequence/NEWALIGN/align.html). Mismatches between the indicated primary isolate Rev amino acid sequence and the consensus subtype C Rev sequence are shown with vertical bars colored by the residue change or gap. Accession numbers are provided as an example of one primary isolate in which the corresponding Rev sequence is found; other primary isolates may also share the same sequence. Sequences are noted as occurring in only donor (D), only recipient (R), or both donor and recipient (B) individuals within a transmission pair. The transmission pair from which the isolate was sequenced is indicated by labeled vertical black and gray bars to the left and right of the sequences. No Rev sequences occurred in multiple transmission pairs. Amino acid positions corresponding to functional domains of Rev are indicated by the boxes at the bottom of the figure. Functional domains are colored for clarity. OD – oligomerization domain; ARM – arginine rich motif; NES – nuclear export signal.

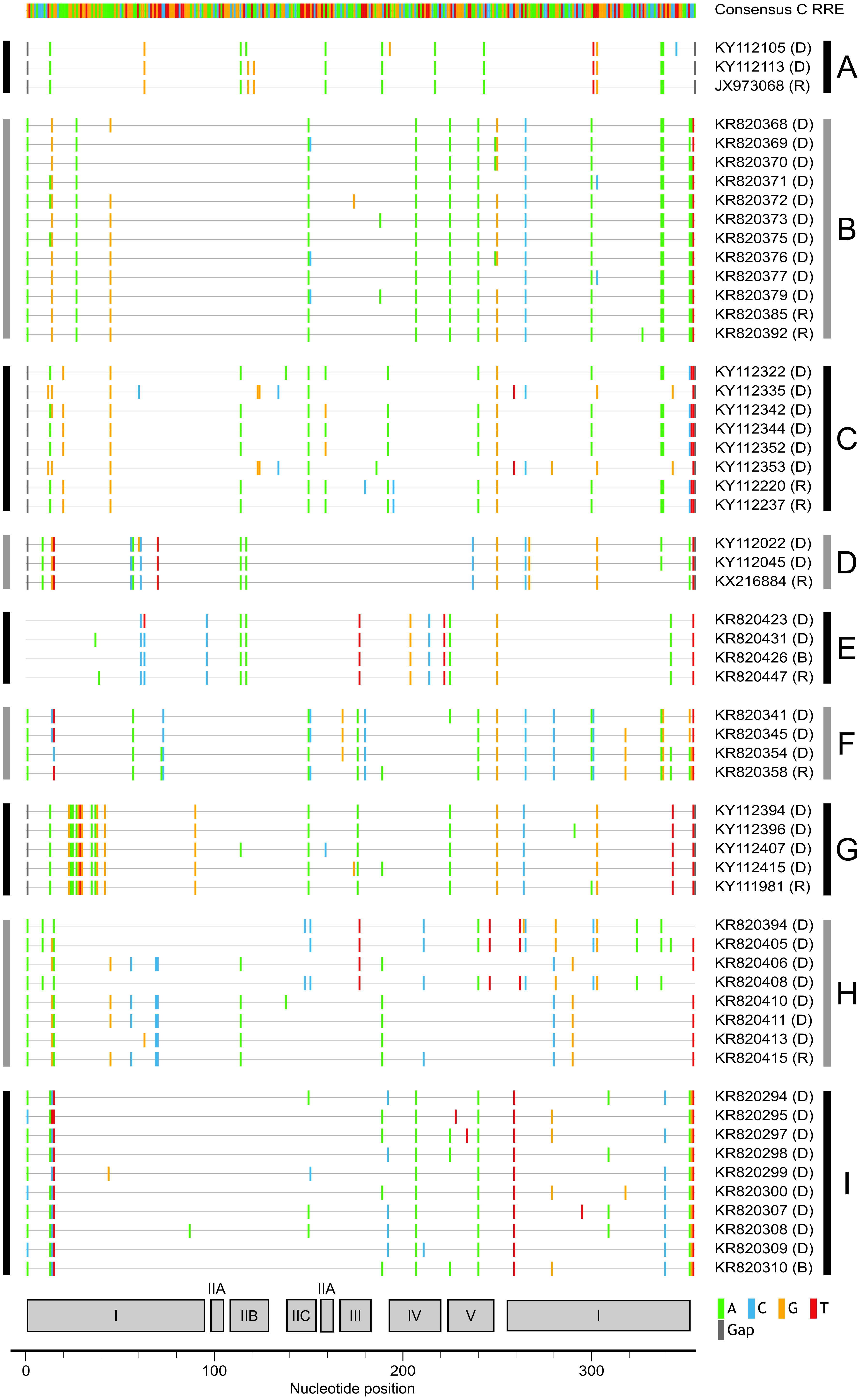

Figure S4. Alignment of unique RRE sequences included in functional assays. Fifty-seven unique RRE nucleotide sequences from primary isolates that were included in functional assays were aligned along with the RRE from a subtype C consensus sequence (Los Alamos HIV database 2002 consensus, https://www.hiv.lanl.gov/content/sequence/NEWALIGN/align.html). Mismatches between the indicated primary isolate RRE sequence and the consensus subtype C RRE sequence are shown with vertical bars colored by the nucleotide change or gap. Accession numbers are provided as an example of one primary isolate in which the corresponding RRE sequence is found; other primary isolates may also share the same sequence. Sequences are noted as occurring in only donor (D), only recipient (R), or both donor and recipient (B) individuals within a transmission pair. The transmission pair from which the isolate was sequenced is indicated by labeled vertical black and gray bars to the left and right of the sequences. No RRE sequences occurred in multiple transmission pairs. Gray boxes at the bottom of the figure indicate nucleotide positions that correspond to the specified stem-loop (I through V) in the NL4-3 five stem-loop structure.

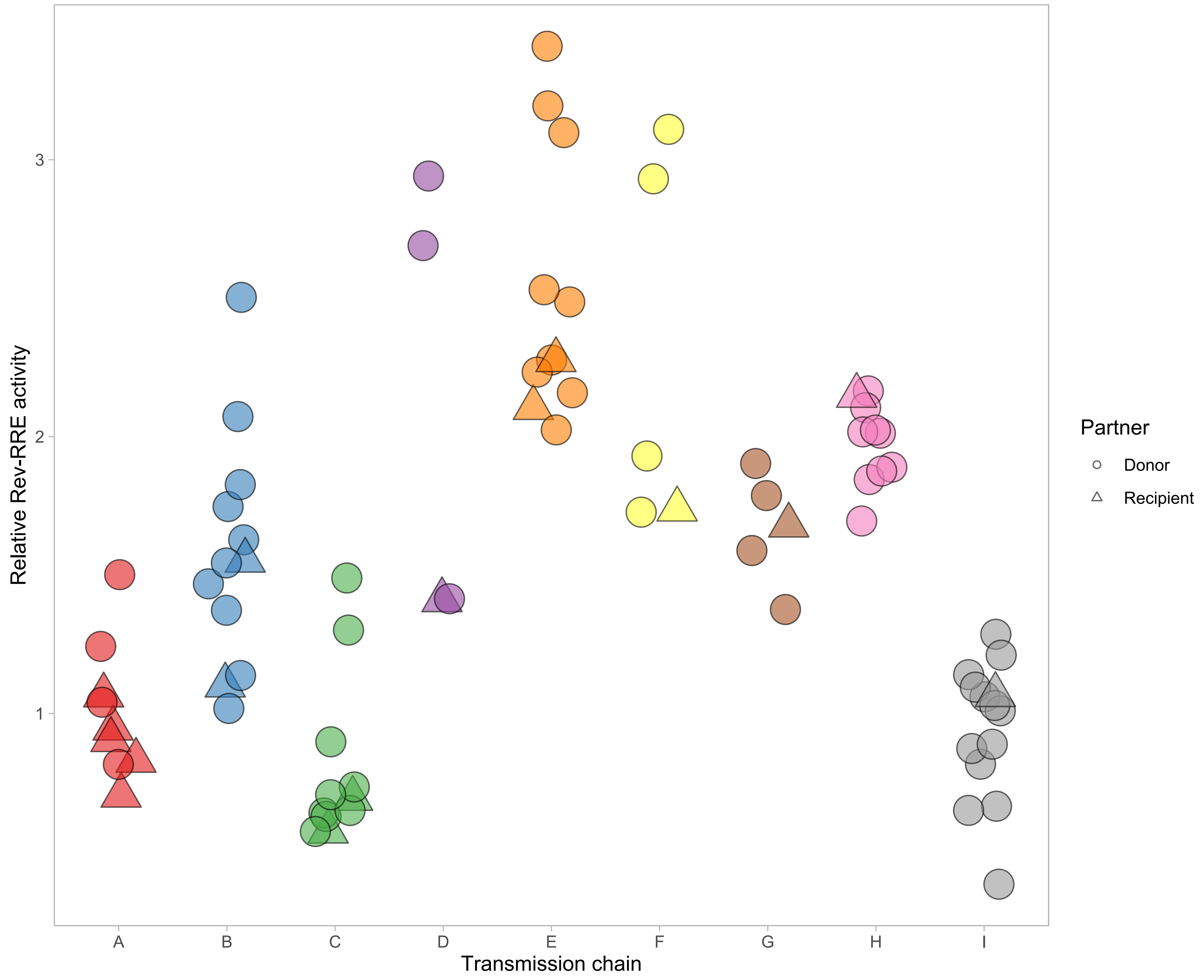

Figure S5. Rev-RRE functional activity of viral variants across transmission chains. Relative Rev-RRE functional activity of all pairs visualized in Figure 2 is again presented using a consistent y-axis for ease of comparison across the entire set. Rev-RRE pairs occurring within different transmission chains, A-I, are separated along the x axis and by color. Each symbol represents a unique Rev-RRE pair in a single individual. Relative activity is indicated on the y-axis in multiples of the functional activity of the NL4-3 Rev-RRE cognate pair. Rev-RRE sequences from donors or recipients are indicated by shape. Unlike in Figure 2, symbols are not sized based on the frequency of the Rev-RRE sequence within a quasispecies.

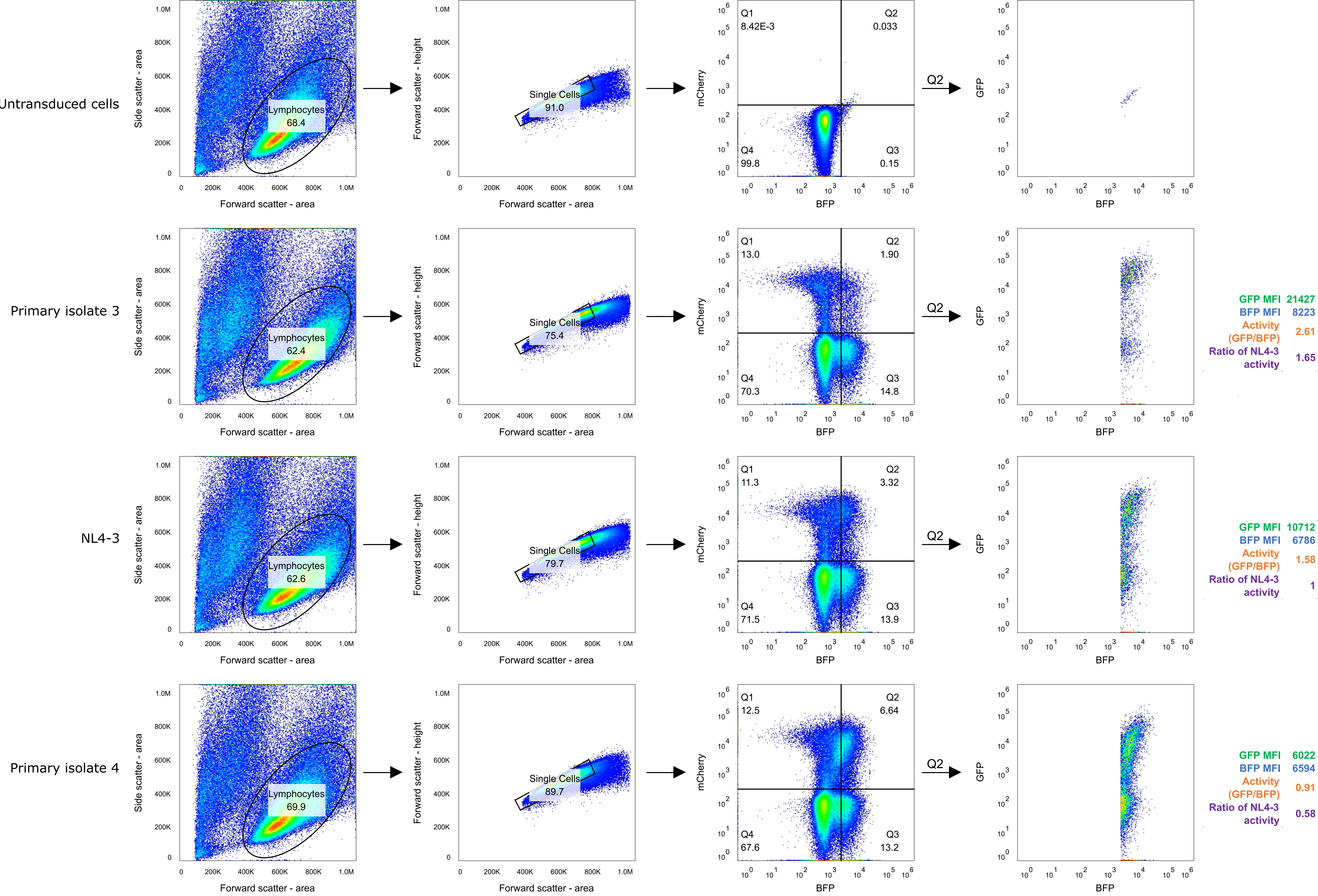

Figure S6. Rev-RRE functional activity assay analysis. An example of the flow cytometry gating strategy and data analysis used for the functional activity assay are shown. From top to bottom are untransduced cells, cells transduced with a lower-activity Rev-RRE pair (from primary isolate 3 shown in Figure 4), cells transduced with the reference NL4-3 Rev-RRE pair, and cells transduced with a higher-activity Rev-RRE pair (from primary isolate 4 shown in Figure 4). The gating strategy for the flow cytometry data proceeds from left to right. First, lymphocytes (i.e. SupT1 cells) are identified in the forward scatter versus side scatter plot and distinguished from cell aggregates and debris. Next, singlet cells are selected. From this population, a quadrant plot is created to identify cells which are successfully transduced with the RRE-containing assay construct (mCherry+) and cells transduced with the Rev-containing assay construct (BFP+). Cells that are cotransduced express both mCherry and BFP and fall into quadrant 2. Finally, in the co-transduced single cell population, the mean fluorescent intensity (MFI) of green fluorescent protein (GFP) and blue florescent protein (BFP) is determined. Rev-RRE activity is calculated as the ratio of GFP and BFP. This activity can be expressed as multiples of the activity of the NL4-3 Rev-RRE cognate pair. In this worked example, the proportion of cells shown in each plot that fall within the indicated gate is shown. Activity calculation was not performed for the untransduced cells as the RRE- and Rev-containing assay constructs are not present.
