# Supplementary material for: Rev–Rev Response Element Activity Selection Bias at the Human Immunodeficiency Virus Transmission Bottleneck
Source: Open Forum Infect Dis. 2023 Sep 29;10(10):ofad486. doi: 10.1093/ofid/ofad486 (PMC10580148; doi:10.1093/ofid/ofad486)
Supplement: ofad486_Supplementary_Data [file ofad486_supplementary_data.zip › Table S1.pdf]

Tabel S1. The Rev-RRE pairs and their relative proportion within the host quasispecies are listed for each individual included in this study. Rev amino acid and RRE nucleotide sequences were given a unique code within an individual participant For example, the Rev-RRE pair "A-B" refers to Rev A with RRE B in the scheme for the indicated individual. An example accession number is provided representing one isolate in which the Rev amino acid and RRE nucleotide sequence pair occurs. Additional isolates may share the same Rev-RRE pair. Relative functional activity values are multiples of the NL4-3 Rev-RRE cognate pair. Mean activity values are the weighted average of the tested pairs. NT - not tested.

**Transmission pair A**									
Donor (CH0212)					Recipient (CH0162)				
**Rev-RRE pair**	**Number of sequences**	**Proportion of sequences**	**Example accession number**	**Relative functional activity**	**Rev-RRE pair**	**Number of sequences**	**Proportion of sequences**	**Example accession number**	**Relative functional activity**
A-B	2	10.00%	KY112105	0.82	A-A	16	27.12%	JX973049	0.95
A-C	1	5.00%	KY112100	NT	A-B	2	3.39%	JX973061	NT
A-D	1	5.00%	KY112097	NT	A-C	1	1.69%	JX973060	NT
A-J	1	5.00%	KY112098	NT	A-D	1	1.69%	JX973066	NT
A-K	1	5.00%	KY112099	NT	A-E	1	1.69%	JX973064	NT
A-L	1	5.00%	KY112104	NT	B-A	18	30.51%	JX972986	0.90
B-A	1	5.00%	KY112119	1.04	B-G	1	1.69%	JX973027	NT
B-F	1	5.00%	KY112111	NT	C-A	8	13.56%	JX973019	1.07
B-H	1	5.00%	KY112108	NT	C-F	1	1.69%	JX973048	NT
B-I	1	5.00%	KY112095	NT	D-A	4	6.78%	JX973043	0.70
C-A	2	10.00%	KY112102	1.24	E-A	3	5.08%	JX973065	0.83
D-A	2	10.00%	KY112113	1.50	F-A	1	1.69%	JX972995	NT
E-B	1	5.00%	KY112101	NT	G-A	1	1.69%	JX972998	NT
F-E	1	5.00%	KY112103	NT	H-A	1	1.69%	JX973025	NT
G-G	1	5.00%	KY112106	NT	**Total**	**59**	**100.00%**	**Mean**	**0.92**
H-A	1	5.00%	KY112107	NT					
I-A	1	5.00%	KY112126	NT					
**Total**	**20**	**100.00%**	**Mean**	**1.17**					

**Transmission pair B**									
Donor (Z3678F)					Recipient (Z3678M)				
**Rev-RRE pair**	**Number of sequences**	**Proportion of sequences**	**Example accession number**	**Relative functional activity**	**Rev-RRE pair**	**Number of sequences**	**Proportion of sequences**	**Example accession number**	**Relative functional activity**
A-A	2	11.11%	KR820369	1.75	A-A	8	88.89%	KR820385	1.10
A-D	1	5.56%	KR820379	1.54	A-B	1	11.11%	KR820392	1.55
A-L	1	5.56%	KR820370	1.37	**Total**	**9**	**100.00%**	**Mean**	**1.15**
A-M	1	5.56%	KR820381	NT					
A-N	1	5.56%	KR820374	NT					
A-P	1	5.56%	KR820384	NT					
B-C	1	5.56%	KR820372	1.02					
B-K	1	5.56%	KR820383	NT					
B-O	1	5.56%	KR820375	1.63					
C-F	1	5.56%	KR820376	2.50					
C-I	1	5.56%	KR820373	1.47					
D-E	1	5.56%	KR820368	2.07					
E-G	1	5.56%	KR820380	NT					
F-H	1	5.56%	KR820371	1.14					
G-B	1	5.56%	KR820377	1.83					

H-J	1	5.56%	KR820382	NT
I-B	1	5.56%	KR820367	NT
**Total**	**18**	**100.00%**	**Mean**	**1.64**

Transmission pair C

Donor (CH0492)				
**Rev-RRE pair**	**Number of sequences**	**Proportion of sequences**	**Example accession number**	**Relative functional activity**
A-A	13	34.21%	KY112346	0.64
A-B	2	5.26%	KY112322	0.63
A-C	2	5.26%	KY112351	0.65
A-E	1	2.63%	KY112356	NT
A-I	1	2.63%	KY112349	NT
A-L	1	2.63%	KY112331	NT
B-D	1	2.63%	KY112343	NT
B-F	1	2.63%	KY112335	1.49
B-O	1	2.63%	KY112334	NT
C-D	1	2.63%	KY112355	NT
C-G	1	2.63%	KY112353	0.57
D-A	2	5.26%	KY112325	0.73
E-A	1	2.63%	KY112326	1.30
F-H	1	2.63%	KY112345	NT
G-P	1	2.63%	KY112337	NT
H-K	1	2.63%	KY112333	NT
I-N	1	2.63%	KY112357	NT
J-J	1	2.63%	KY112348	NT
K-A	1	2.63%	KY112336	NT
L-A	1	2.63%	KY112332	NT
M-A	1	2.63%	KY112350	NT
N-A	1	2.63%	KY112339	0.90
O-M	1	2.63%	KY112342	0.71
**Total**	**38**	**100.00%**	**Mean**	**0.72**

Recipient (CH0427)				
**Rev-RRE pair**	**Number of sequences**	**Proportion of sequences**	**Example accession number**	**Relative functional activity**
A-A	27	81.82%	KY112218	0.69
A-B	2	6.06%	KY112229	0.57
A-C	1	3.03%	KY112250	NT
A-D	1	3.03%	KY112247	NT
A-E	1	3.03%	KY112245	NT
B-A	1	3.03%	KY112224	NT
**Total**	**33**	**100.00%**	**Mean**	**0.68**

Transmission pair D

Donor (CH1064)				
**Rev-RRE pair**	**Number of sequences**	**Proportion of sequences**	**Example accession number**	**Relative functional activity**
A-A	19	46.34%	KY112016	2.94
A-B	3	7.32%	KY112022	2.69
A-C	1	2.44%	KY112034	NT
A-G	1	2.44%	KY112023	NT
A-H	1	2.44%	KY112044	NT
A-I	1	2.44%	KY112021	NT
B-A	8	19.51%	KY112045	1.41
B-B	1	2.44%	KY112018	NT
B-D	1	2.44%	KY112031	NT
B-F	1	2.44%	KY112047	NT
C-A	1	2.44%	KY112032	NT
D-A	1	2.44%	KY112040	NT
E-E	1	2.44%	KY112046	NT

Recipient (CH0848)				
**Rev-RRE pair**	**Number of sequences**	**Proportion of sequences**	**Example accession number**	**Relative functional activity**
A-A	9	75.00%	KX216884	1.41
A-B	1	8.33%	KX216892	NT
A-C	1	8.33%	KX216889	NT
B-A	1	8.33%	KX216895	NT
**Total**	**12**	**100.00%**	**Mean**	**1.41**

F-A	1	2.44%	KY112052	NT
**Total**	**41**	**100.00%**	**Mean**	**2.51**

Transmission pair E

Donor (Z4473F)				
**Rev-RRE pair**	**Number of sequences**	**Proportion of sequences**	**Example accession number**	**Relative functional activity**
A-A	1	5.56%	KR820422	NT
B-B	3	16.67%	KR820423	3.41
C-A	2	11.11%	KR820426	2.49
C-B	1	5.56%	KR820436	2.53
C-C	3	16.67%	KR820431	3.10
D-A	2	11.11%	KR820427	2.28
E-A	1	5.56%	KR820429	2.02
F-A	1	5.56%	KR820438	2.16
F-C	1	5.56%	KR820430	2.23
G-B	1	5.56%	KR820435	3.19
G-D	1	5.56%	KR820433	NT
H-B	1	5.56%	KR820434	NT
**Total**	**18**	**100.00%**	**Mean**	**2.75**

Recipient (Z4473M)				
**Rev-RRE pair**	**Number of sequences**	**Proportion of sequences**	**Example accession number**	**Relative functional activity**
A-A	9	90.00%	KR820440	2.28
A-B	1	10.00%	KR820447	2.10
**Total**	**10**	**100.00%**	**Mean**	**2.26**

Transmission pair F

Donor (Z3618F)				
**Rev-RRE pair**	**Number of sequences**	**Proportion of sequences**	**Example accession number**	**Relative functional activity**
A-A	4	23.53%	KR820350	3.11
A-C	1	5.88%	KR820349	NT
B-A	2	11.76%	KR820343	2.93
B-B	1	5.88%	KR820351	1.93
B-G	1	5.88%	KR820348	NT
C-D	1	5.88%	KR820356	NT
C-E	1	5.88%	KR820354	1.73
C-F	1	5.88%	KR820342	NT
D-B	1	5.88%	KR820345	NT
E-A	1	5.88%	KR820341	NT
F-A	1	5.88%	KR820353	NT
G-A	1	5.88%	KR820346	NT
H-H	1	5.88%	KR820347	NT
**Total**	**17**	**100.00%**	**Mean**	**2.74**

Recipient (Z3618M)				
**Rev-RRE pair**	**Number of sequences**	**Proportion of sequences**	**Example accession number**	**Relative functional activity**
A-A	7	77.78%	KR820358	1.74
A-B	1	11.11%	KR820365	NT
B-A	1	11.11%	KR820360	NT
**Total**	**9**	**100.00%**	**Mean**	**1.74**

Transmission pair G

Donor (CH0596)				
**Rev-RRE pair**	**Number of sequences**	**Proportion of sequences**	**Example accession number**	**Relative functional activity**
A-A	4	10.26%	KY112428	1.59
A-AA	1	2.56%	KY112426	NT
A-C	2	5.13%	KY112396	1.38
A-D	2	5.13%	KY112391	1.79
A-F	1	2.56%	KY112423	NT

Recipient (CH0455)				
**Rev-RRE pair**	**Number of sequences**	**Proportion of sequences**	**Example accession number**	**Relative functional activity**
A-A	15	83.33%	KY111965	1.68
A-B	1	5.56%	KY111966	NT
B-A	1	5.56%	KY111981	NT
C-A	1	5.56%	KY111982	NT
**Total**	**18**	**100.00%**	**Mean**	**1.68**

A-J	1	2.56%	KY112424	NT
A-K	1	2.56%	KY112416	NT
B-A	1	2.56%	KY112392	NT
B-B	2	5.13%	KY112407	1.90
B-C	1	2.56%	KY112419	NT
B-I	1	2.56%	KY112408	NT
B-M	1	2.56%	KY112404	NT
C-A	1	2.56%	KY112425	NT
C-B	1	2.56%	KY112410	NT
C-E	1	2.56%	KY112412	NT
C-G	1	2.56%	KY112421	NT
C-H	1	2.56%	KY112403	NT
C-P	1	2.56%	KY112402	NT
D-S	1	2.56%	KY112409	NT
D-W	1	2.56%	KY112395	NT
D-X	1	2.56%	KY112414	NT
D-Y	1	2.56%	KY112390	NT
E-Q	1	2.56%	KY112398	NT
E-U	1	2.56%	KY112399	NT
F-R	1	2.56%	KY112401	NT
F-V	1	2.56%	KY112397	NT
G-Z	1	2.56%	KY112422	NT
H-O	1	2.56%	KY112405	NT
I-B	1	2.56%	KY112418	NT
J-L	1	2.56%	KY112413	NT
K-E	1	2.56%	KY112417	NT
L-N	1	2.56%	KY112420	NT
M-T	1	2.56%	KY112400	NT
**Total**	**39**	**100.00%**	**Mean**	**1.65**

Transmission pair H

Donor (Z4248F)				
**Rev-RRE pair**	**Number of sequences**	**Proportion of sequences**	**Example accession number**	**Relative functional activity**
A-A	1	4.76%	KR820414	NT
A-H	1	4.76%	KR820405	2.17
B-B	3	14.29%	KR820413	1.85
B-D	1	4.76%	KR820411	2.10
B-L	1	4.76%	KR820396	NT
B-M	1	4.76%	KR820395	NT
C-C	1	4.76%	KR820412	NT
D-D	1	4.76%	KR820403	1.89
D-E	1	4.76%	KR820410	2.01
D-G	4	19.05%	KR820406	1.88
E-F	1	4.76%	KR820408	2.02
E-K	1	4.76%	KR820398	NT
F-F	1	4.76%	KR820407	1.70
F-I	1	4.76%	KR820404	NT
G-J	1	4.76%	KR820401	NT
H-N	1	4.76%	KR820394	2.02
**Total**	**21**	**100.00%**	**Mean**	**1.92**

Recipient (Z4248M)				
**Rev-RRE pair**	**Number of sequences**	**Proportion of sequences**	**Example accession number**	**Relative functional activity**
A-A	7	100.00%	KR820421	2.15
**Total**	**7**	**100.00%**	**Mean**	**2.15**

Transmission pair I

Donor (Z331F)

Rev-RRE pair	Number of sequences	Proportion of sequences	Example accession number	Relative functional activity
A-B	2	10.00%	KR820310	1.14
A-C	2	10.00%	KR820300	1.06
A-K	1	5.00%	KR820297	1.01
B-A	1	5.00%	KR820294	0.67
B-D	1	5.00%	KR820299	1.10
B-F	1	5.00%	KR820308	0.65
B-H	1	5.00%	KR820304	NT
B-J	1	5.00%	KR820298	0.82
C-A	3	15.00%	KR820301	0.87
C-I	1	5.00%	KR820307	0.38
D-A	2	10.00%	KR820302	0.89
E-B	1	5.00%	KR820313	1.29
E-E	1	5.00%	KR820309	1.03
F-A	1	5.00%	KR820296	NT
G-G	1	5.00%	KR820295	1.21
**Total**	**20**	**100.00%**	**Mean**	**0.94**

Recipient (Z331M)

Rev-RRE pair	Number of sequences	Proportion of sequences	Example accession number	Relative functional activity
A-A	9	90.00%	KR820314	1.07
B-A	1	10.00%	KR820320	NT
**Total**	**10**	**100.00%**	**Mean**	**1.07**
