# Supplementary material for: Rev–Rev Response Element Activity Selection Bias at the Human Immunodeficiency Virus Transmission Bottleneck
Source: Open Forum Infect Dis. 2023 Sep 29;10(10):ofad486. doi: 10.1093/ofid/ofad486 (PMC10580148; doi:10.1093/ofid/ofad486)
Supplement: ofad486_Supplementary_Data [file ofad486_supplementary_data.zip › Table S2.pdf]

Table S2. Accession numbers of viral sequences utilized in this study are listed below. (One viral sequence for subject CH0162 [JX973054] contained a premature stop codon within *rev* and was not included in the analysis of Rev-RRE cognate pairs.)

Genome accession number	Subject ID	Rev-RRE pair	Rev-RRE included in functional assay
JX972986	CH0162	B-A	Yes
JX972987	CH0162	B-A	Yes
JX972988	CH0162	B-A	Yes
JX972989	CH0162	B-A	Yes
JX972990	CH0162	B-A	Yes
JX972991	CH0162	B-A	Yes
JX972992	CH0162	B-A	Yes
JX972993	CH0162	B-A	Yes
JX972994	CH0162	B-A	Yes
JX972995	CH0162	F-A	No
JX972996	CH0162	B-A	Yes
JX972997	CH0162	B-A	Yes
JX972998	CH0162	G-A	No
JX973019	CH0162	C-A	Yes
JX973020	CH0162	B-A	Yes
JX973021	CH0162	C-A	Yes
JX973022	CH0162	B-A	Yes
JX973023	CH0162	C-A	Yes
JX973024	CH0162	B-A	Yes
JX973025	CH0162	H-A	No
JX973026	CH0162	B-A	Yes
JX973027	CH0162	B-G	No
JX973028	CH0162	B-A	Yes
JX973029	CH0162	C-A	Yes
JX973030	CH0162	C-A	Yes
JX973031	CH0162	B-A	Yes
JX973032	CH0162	B-A	Yes
JX973033	CH0162	C-A	Yes
JX973043	CH0162	D-A	Yes
JX973044	CH0162	C-A	Yes
JX973045	CH0162	D-A	Yes
JX973046	CH0162	D-A	Yes
JX973047	CH0162	D-A	Yes
JX973048	CH0162	C-F	No
JX973049	CH0162	A-A	Yes
JX973050	CH0162	C-A	Yes
JX973051	CH0162	A-A	Yes
JX973052	CH0162	A-A	Yes
JX973053	CH0162	A-A	Yes
JX973055	CH0162	A-A	Yes
JX973056	CH0162	A-A	Yes
JX973057	CH0162	A-A	Yes

JX973058	CH0162	A-A	Yes
JX973059	CH0162	A-A	Yes
JX973060	CH0162	A-C	No
JX973061	CH0162	A-B	No
JX973062	CH0162	A-A	Yes
JX973063	CH0162	A-A	Yes
JX973064	CH0162	A-E	No
JX973065	CH0162	E-A	Yes
JX973066	CH0162	A-D	No
JX973067	CH0162	E-A	Yes
JX973068	CH0162	E-A	Yes
JX973069	CH0162	A-A	Yes
JX973070	CH0162	A-A	Yes
JX973071	CH0162	A-A	Yes
JX973072	CH0162	A-A	Yes
JX973073	CH0162	A-B	No
JX973074	CH0162	A-A	Yes
KR820294	Z331F	B-A	Yes
KR820295	Z331F	G-G	Yes
KR820296	Z331F	F-A	No
KR820297	Z331F	A-K	Yes
KR820298	Z331F	B-J	Yes
KR820299	Z331F	B-D	Yes
KR820300	Z331F	A-C	Yes
KR820301	Z331F	C-A	Yes
KR820302	Z331F	D-A	Yes
KR820303	Z331F	C-A	Yes
KR820304	Z331F	B-H	No
KR820305	Z331F	A-C	Yes
KR820306	Z331F	C-A	Yes
KR820307	Z331F	C-I	Yes
KR820308	Z331F	B-F	Yes
KR820309	Z331F	E-E	Yes
KR820310	Z331F	A-B	Yes
KR820311	Z331F	A-B	Yes
KR820312	Z331F	D-A	Yes
KR820313	Z331F	E-B	Yes
KR820314	Z331M	A-A	Yes
KR820315	Z331M	A-A	Yes
KR820316	Z331M	A-A	Yes
KR820317	Z331M	A-A	Yes
KR820318	Z331M	A-A	Yes
KR820319	Z331M	A-A	Yes
KR820320	Z331M	B-A	No
KR820321	Z331M	A-A	Yes
KR820322	Z331M	A-A	Yes
KR820323	Z331M	A-A	Yes

KR820341	Z3618F	E-A	No
KR820342	Z3618F	C-F	No
KR820343	Z3618F	B-A	Yes
KR820344	Z3618F	B-A	Yes
KR820345	Z3618F	D-B	No
KR820346	Z3618F	G-A	No
KR820347	Z3618F	H-H	No
KR820348	Z3618F	B-G	No
KR820349	Z3618F	A-C	No
KR820350	Z3618F	A-A	Yes
KR820351	Z3618F	B-B	Yes
KR820352	Z3618F	A-A	Yes
KR820353	Z3618F	F-A	No
KR820354	Z3618F	C-E	Yes
KR820355	Z3618F	A-A	Yes
KR820356	Z3618F	C-D	No
KR820357	Z3618F	A-A	Yes
KR820358	Z3618M	A-A	Yes
KR820359	Z3618M	A-A	Yes
KR820360	Z3618M	B-A	No
KR820361	Z3618M	A-A	Yes
KR820362	Z3618M	A-A	Yes
KR820363	Z3618M	A-A	Yes
KR820364	Z3618M	A-A	Yes
KR820365	Z3618M	A-B	No
KR820366	Z3618M	A-A	Yes
KR820367	Z3678F	I-B	No
KR820368	Z3678F	D-E	Yes
KR820369	Z3678F	A-A	Yes
KR820370	Z3678F	A-L	Yes
KR820371	Z3678F	F-H	Yes
KR820372	Z3678F	B-C	Yes
KR820373	Z3678F	C-I	Yes
KR820374	Z3678F	A-N	No
KR820375	Z3678F	B-O	Yes
KR820376	Z3678F	C-F	Yes
KR820377	Z3678F	G-B	Yes
KR820378	Z3678F	A-A	Yes
KR820379	Z3678F	A-D	Yes
KR820380	Z3678F	E-G	No
KR820381	Z3678F	A-M	No
KR820382	Z3678F	H-J	No
KR820383	Z3678F	B-K	No
KR820384	Z3678F	A-P	No
KR820385	Z3678M	A-A	Yes
KR820386	Z3678M	A-A	Yes
KR820387	Z3678M	A-A	Yes

KR820388	Z3678M	A-A	Yes
KR820389	Z3678M	A-A	Yes
KR820390	Z3678M	A-A	Yes
KR820391	Z3678M	A-A	Yes
KR820392	Z3678M	A-B	Yes
KR820393	Z3678M	A-A	Yes
KR820394	Z4248F	H-N	Yes
KR820395	Z4248F	B-M	No
KR820396	Z4248F	B-L	No
KR820397	Z4248F	D-G	Yes
KR820398	Z4248F	E-K	No
KR820399	Z4248F	B-B	Yes
KR820400	Z4248F	D-G	Yes
KR820401	Z4248F	G-J	No
KR820402	Z4248F	D-G	Yes
KR820403	Z4248F	D-D	Yes
KR820404	Z4248F	F-I	No
KR820405	Z4248F	A-H	Yes
KR820406	Z4248F	D-G	Yes
KR820407	Z4248F	F-F	Yes
KR820408	Z4248F	E-F	Yes
KR820409	Z4248F	B-B	Yes
KR820410	Z4248F	D-E	Yes
KR820411	Z4248F	B-D	Yes
KR820412	Z4248F	C-C	No
KR820413	Z4248F	B-B	Yes
KR820414	Z4248F	A-A	No
KR820415	Z4248M	A-A	Yes
KR820416	Z4248M	A-A	Yes
KR820417	Z4248M	A-A	Yes
KR820418	Z4248M	A-A	Yes
KR820419	Z4248M	A-A	Yes
KR820420	Z4248M	A-A	Yes
KR820421	Z4248M	A-A	Yes
KR820422	Z4473F	A-A	No
KR820423	Z4473F	B-B	Yes
KR820424	Z4473F	B-B	Yes
KR820425	Z4473F	B-B	Yes
KR820426	Z4473F	C-A	Yes
KR820427	Z4473F	D-A	Yes
KR820428	Z4473F	D-A	Yes
KR820429	Z4473F	E-A	Yes
KR820430	Z4473F	F-C	Yes
KR820431	Z4473F	C-C	Yes
KR820432	Z4473F	C-A	Yes
KR820433	Z4473F	G-D	No
KR820434	Z4473F	H-B	No

KR820435	Z4473F	G-B	Yes
KR820436	Z4473F	C-B	Yes
KR820437	Z4473F	C-C	Yes
KR820438	Z4473F	F-A	Yes
KR820439	Z4473F	C-C	Yes
KR820440	Z4473M	A-A	Yes
KR820441	Z4473M	A-A	Yes
KR820442	Z4473M	A-A	Yes
KR820443	Z4473M	A-A	Yes
KR820444	Z4473M	A-A	Yes
KR820445	Z4473M	A-A	Yes
KR820446	Z4473M	A-A	Yes
KR820447	Z4473M	A-B	Yes
KR820448	Z4473M	A-A	Yes
KR820449	Z4473M	A-A	Yes
KX216883	CH0848	A-A	Yes
KX216884	CH0848	A-A	Yes
KX216885	CH0848	A-A	Yes
KX216886	CH0848	A-A	Yes
KX216887	CH0848	A-A	Yes
KX216888	CH0848	A-A	Yes
KX216889	CH0848	A-C	No
KX216890	CH0848	A-A	Yes
KX216891	CH0848	A-A	Yes
KX216892	CH0848	A-B	No
KX216893	CH0848	A-A	Yes
KX216895	CH0848	B-A	No
KY111965	CH0455	A-A	Yes
KY111966	CH0455	A-B	No
KY111967	CH0455	A-A	Yes
KY111968	CH0455	A-A	Yes
KY111969	CH0455	A-A	Yes
KY111970	CH0455	A-A	Yes
KY111971	CH0455	A-A	Yes
KY111972	CH0455	A-A	Yes
KY111973	CH0455	A-A	Yes
KY111974	CH0455	A-A	Yes
KY111975	CH0455	A-A	Yes
KY111976	CH0455	A-A	Yes
KY111977	CH0455	A-A	Yes
KY111978	CH0455	A-A	Yes
KY111979	CH0455	A-A	Yes
KY111980	CH0455	A-A	Yes
KY111981	CH0455	B-A	No
KY111982	CH0455	C-A	No
KY112015	CH1064	A-A	Yes
KY112016	CH1064	A-A	Yes

KY112017	CH1064	A-A	Yes
KY112018	CH1064	B-B	No
KY112019	CH1064	B-A	Yes
KY112020	CH1064	A-B	Yes
KY112021	CH1064	A-I	No
KY112022	CH1064	A-B	Yes
KY112023	CH1064	A-G	No
KY112024	CH1064	B-A	Yes
KY112025	CH1064	A-A	Yes
KY112026	CH1064	B-A	Yes
KY112027	CH1064	A-A	Yes
KY112028	CH1064	B-A	Yes
KY112029	CH1064	A-A	Yes
KY112030	CH1064	A-A	Yes
KY112031	CH1064	B-D	No
KY112032	CH1064	C-A	No
KY112033	CH1064	A-A	Yes
KY112034	CH1064	A-C	No
KY112035	CH1064	A-A	Yes
KY112036	CH1064	A-A	Yes
KY112037	CH1064	B-A	Yes
KY112038	CH1064	A-A	Yes
KY112039	CH1064	A-A	Yes
KY112040	CH1064	D-A	No
KY112041	CH1064	A-A	Yes
KY112042	CH1064	A-A	Yes
KY112043	CH1064	A-A	Yes
KY112044	CH1064	A-H	No
KY112045	CH1064	B-A	Yes
KY112046	CH1064	E-E	No
KY112047	CH1064	B-F	No
KY112048	CH1064	A-A	Yes
KY112049	CH1064	B-A	Yes
KY112050	CH1064	A-B	Yes
KY112051	CH1064	A-A	Yes
KY112052	CH1064	F-A	No
KY112053	CH1064	A-A	Yes
KY112054	CH1064	A-A	Yes
KY112055	CH1064	B-A	Yes
KY112094	CH0212	D-A	Yes
KY112095	CH0212	B-I	No
KY112096	CH0212	C-A	Yes
KY112097	CH0212	A-D	No
KY112098	CH0212	A-J	No
KY112099	CH0212	A-K	No
KY112100	CH0212	A-C	No
KY112101	CH0212	E-B	No

KY112102	CH0212	C-A	Yes
KY112103	CH0212	F-E	No
KY112104	CH0212	A-L	No
KY112105	CH0212	A-B	Yes
KY112106	CH0212	G-G	No
KY112107	CH0212	H-A	No
KY112108	CH0212	B-H	No
KY112111	CH0212	B-F	No
KY112113	CH0212	D-A	Yes
KY112119	CH0212	B-A	Yes
KY112125	CH0212	A-B	Yes
KY112126	CH0212	I-A	No
KY112218	CH0427	A-A	Yes
KY112219	CH0427	A-A	Yes
KY112220	CH0427	A-B	Yes
KY112221	CH0427	A-A	Yes
KY112222	CH0427	A-A	Yes
KY112223	CH0427	A-A	Yes
KY112224	CH0427	B-A	No
KY112225	CH0427	A-A	Yes
KY112226	CH0427	A-A	Yes
KY112227	CH0427	A-A	Yes
KY112228	CH0427	A-A	Yes
KY112229	CH0427	A-B	Yes
KY112230	CH0427	A-A	Yes
KY112231	CH0427	A-A	Yes
KY112232	CH0427	A-A	Yes
KY112233	CH0427	A-A	Yes
KY112234	CH0427	A-A	Yes
KY112235	CH0427	A-A	Yes
KY112236	CH0427	A-A	Yes
KY112237	CH0427	A-A	Yes
KY112238	CH0427	A-A	Yes
KY112239	CH0427	A-A	Yes
KY112240	CH0427	A-A	Yes
KY112241	CH0427	A-A	Yes
KY112242	CH0427	A-A	Yes
KY112243	CH0427	A-A	Yes
KY112244	CH0427	A-A	Yes
KY112245	CH0427	A-E	No
KY112246	CH0427	A-A	Yes
KY112247	CH0427	A-D	No
KY112248	CH0427	A-A	Yes
KY112249	CH0427	A-A	Yes
KY112250	CH0427	A-C	No
KY112322	CH0492	A-B	Yes
KY112323	CH0492	A-A	Yes

KY112324	CH0492	A-A	Yes
KY112325	CH0492	D-A	Yes
KY112326	CH0492	E-A	Yes
KY112327	CH0492	A-A	Yes
KY112328	CH0492	A-A	Yes
KY112329	CH0492	A-A	Yes
KY112330	CH0492	A-A	Yes
KY112331	CH0492	A-L	No
KY112332	CH0492	L-A	No
KY112333	CH0492	H-K	No
KY112334	CH0492	B-O	No
KY112335	CH0492	B-F	Yes
KY112336	CH0492	K-A	No
KY112337	CH0492	G-P	No
KY112338	CH0492	D-A	Yes
KY112339	CH0492	N-A	Yes
KY112340	CH0492	A-A	Yes
KY112341	CH0492	A-A	Yes
KY112342	CH0492	O-M	Yes
KY112343	CH0492	B-D	No
KY112344	CH0492	A-A	Yes
KY112345	CH0492	F-H	No
KY112346	CH0492	A-A	Yes
KY112347	CH0492	A-A	Yes
KY112348	CH0492	J-J	No
KY112349	CH0492	A-I	No
KY112350	CH0492	M-A	No
KY112351	CH0492	A-C	Yes
KY112352	CH0492	A-C	Yes
KY112353	CH0492	C-G	Yes
KY112354	CH0492	A-A	Yes
KY112355	CH0492	C-D	No
KY112356	CH0492	A-E	No
KY112357	CH0492	I-N	No
KY112358	CH0492	A-B	Yes
KY112359	CH0492	A-A	Yes
KY112390	CH0596	D-Y	No
KY112391	CH0596	A-D	Yes
KY112392	CH0596	B-A	No
KY112393	CH0596	A-A	Yes
KY112394	CH0596	A-A	Yes
KY112395	CH0596	D-W	No
KY112396	CH0596	A-C	Yes
KY112397	CH0596	F-V	No
KY112398	CH0596	E-Q	No
KY112399	CH0596	E-U	No
KY112400	CH0596	M-T	No

KY112401	CH0596	F-R	No
KY112402	CH0596	C-P	No
KY112403	CH0596	C-H	No
KY112404	CH0596	B-M	No
KY112405	CH0596	H-O	No
KY112406	CH0596	A-C	Yes
KY112407	CH0596	B-B	Yes
KY112408	CH0596	B-I	No
KY112409	CH0596	D-S	No
KY112410	CH0596	C-B	No
KY112411	CH0596	B-B	Yes
KY112412	CH0596	C-E	No
KY112413	CH0596	J-L	No
KY112414	CH0596	D-X	No
KY112415	CH0596	A-D	Yes
KY112416	CH0596	A-K	No
KY112417	CH0596	K-E	No
KY112418	CH0596	I-B	No
KY112419	CH0596	B-C	No
KY112420	CH0596	L-N	No
KY112421	CH0596	C-G	No
KY112422	CH0596	G-Z	No
KY112423	CH0596	A-F	No
KY112424	CH0596	A-J	No
KY112425	CH0596	C-A	No
KY112426	CH0596	A-AA	No
KY112427	CH0596	A-A	Yes
KY112428	CH0596	A-A	Yes
