# Supplementary material for: Rev–Rev Response Element Activity Selection Bias at the Human Immunodeficiency Virus Transmission Bottleneck
Source: Open Forum Infect Dis. 2023 Sep 29;10(10):ofad486. doi: 10.1093/ofid/ofad486 (PMC10580148; doi:10.1093/ofid/ofad486)
Supplement: ofad486_Supplementary_Data [file ofad486_supplementary_data.zip › Table S3.pdf]

Table S3. Plasmid constructs utilized in functional assays are listed. A single Rev or RRE sequence included in a plasmid construct may occur in multiple individuals and thus have multiple sequence designations.

Rev plasmid constructs (pMSCV-Rev-IRES-eBFP2)	
**Rev plasmid number**	**Rev sequence designation**
5864	NL4-3 Rev
6000	CH0596 Rev B
6002	CH0596 Rev A
6004	CH0492 Rev D
6006	CH0492 Rev A
6008	CH0455 Rev A
6010	CH0427 Rev A
6012	CH0212 Rev D
6014	CH0212 Rev C
6016	CH0212 Rev B
6018	CH0212 Rev A
6020	CH0162 Rev E
6022	CH0162 Rev D
6024	CH0162 Rev C
6026	CH0162 Rev B
6028	CH0162 Rev A
6165	Z3678M Rev A
6166	Z3678F Rev B
6167	Z3678F Rev A
6168	Z3618M Rev A/Z3618F Rev C
6169	Z3618F Rev B
6170	Z3618F Rev A
6171	Z331M Rev A
6172	Z331F Rev D
6173	Z331F Rev C
6174	Z331F Rev B
6175	Z331F Rev A
6176	CH1064 Rev A
6177	CH0848 Rev A/CH1064 Rev B
6486	CH0492 Rev B
6487	CH0492 Rev C
6488	CH0492 Rev E
6489	CH0492 Rev N
6490	CH0492 Rev O
6494	Z331F Rev E
6495	Z331F Rev G
6502	Z4248F Rev A
6503	Z4248F Rev B/Z4248M Rev A
6504	Z4248F Rev D
6505	Z4248F Rev E
6506	Z4248F Rev F
6507	Z4248F Rev H
6515	Z4473F Rev B
6516	Z4473F Rev C
6517	Z4473F Rev D/Z4473M Rev A

RRE plasmid constructs [pNL4-3(myr-)(CHYSEL-eGFP)(Rev-)(Vpr-)(Env-)(XmaI-RRE-XbaI)(mCherry+)(Nef-)]	
**RRE plasmid number**	**RRE sequence designation**
5936	NL4- RRE
6030	CH0596 RRE D
6032	CH0596 RRE C
6034	CH0596 RRE B
6036	CH0596 RRE A
6038	CH0492 RRE C
6040	CH0492 RRE B
6042	CH0492 RRE A
6044	CH0455 RRE A
6046	CH0427 RRE B
6048	CH0427 RRE A
6050	CH0212 RRE B
6052	CH0212 RRE A
6054	CH0162 RRE A
6178	Z3678M RRE A
6179	Z3678F RRE O
6180	Z3678F RRE A
6181	Z3618M RRE A
6182	Z3618F RRE E
6183	Z3618F RRE B
6184	Z3618F RRE A
6185	Z331M RRE A/Z331F RRE B
6186	Z331F RRE K
6187	Z331F RRE C
6188	Z331F RRE A
6189	CH1064 RRE B
6190	CH1064 RRE A
6191	CH0848 RRE A
6491	CH0492 RRE F
6492	CH0492 RRE G
6493	CH0492 RRE M
6496	Z331F RRE D
6497	Z331F RRE E
6498	Z331F RRE F
6499	Z331F RRE G
6500	Z331F RRE I
6501	Z331F RRE J
6508	Z4248F RRE B
6509	Z4248F RRE D
6510	Z4248F RRE E
6511	Z4248F RRE F
6512	Z4248F RRE H
6513	Z4248M RRE A
6514	Z4248F RRE G
6521	Z4473F RRE A/Z4473M RRE A

6518	Z4473F Rev E
6519	Z4473F Rev F
6520	Z4473F Rev G
6662	Z3678F Rev G
6663	Z3678F Rev F
6664	Z3678F Rev D
6665	Z3678F Rev C

6522	Z4473F RRE B
6523	Z4473F RRE C
6524	Z4473M RRE B
6670	Z4248F RRE N
6671	Z3678M RRE B
6672	Z3678F RRE L
6673	Z3678F RRE I
6674	Z3678F RRE H
6675	Z3678F RRE F
6676	Z3678F RRE E
6677	Z3678F RRE D
6678	Z3678F RRE C
6679	Z3678F RRE B
